# GB5, a synergistic phytotherapy for type 2 diabetes mellitus management: an integrated polyherbal approach from phytochemical profiling to network pharmacology

**DOI:** 10.1186/s12906-025-05192-3

**Published:** 2025-12-11

**Authors:** Arghadip Das, Narmadha Chinnadurai Rajeswari, Priyadharshini Palani, Navaneedhakrishnan Manigandan, Sujatha Kuppusamy, Gayatri Sukumaran, Gayathri Veeraraghavan, Raman Lakshmi Sundaram

**Affiliations:** 1 Centre for Toxicology and Developmental Research (CEFTE), Sri Ramachandra Institute of Higher Education and Research (DU), Chennai, Tamil Nadu 600116 India; 2 Sri Ramachandra Faculty of Pharmacy, Sri Ramachandra Institute of Higher Education and Research (DU), Chennai, Tamil Nadu 600116 India; 3 Faculty of Clinical Research, Sri Ramachandra Institute of Higher Education and Research (DU), Chennai, Tamil Nadu 600116 India

**Keywords:** Type 2 diabetes mellitus, Polyherbal synergy, Enzyme kinetics, PPAR, Glucose uptake, Lipid accumulation

## Abstract

**Background:**

Type 2 diabetes mellitus (T2DM) remains a global health burden characterized by insulin resistance, persistent hyperglycaemia, and chronic inflammation. Although single-target therapies effectively reduce glucose levels, they seldom address oxidative stress or adipocyte dysfunction. Polyherbal formulations (PHFs) harness synergistic phytochemicals for multimodal intervention; however, many lack mechanistic transparency owing to excessive inclusion of diverse botanical bioactives, suffer from non-standardized composition, and underexplore volatile antidiabetic constituents. Confronting these challenges, we developed Gluco Balance V (GB5), a cold-percolated ethanolic extract comprising equal proportions of bio-effective parts of *Asparagus racemosus*, *Cyperus rotundus*, *Tinospora cordifolia*, *Terminalia arjuna*, and *Mimosa pudica*. These botanicals were selected for complementary antidiabetic, antioxidant, and adiporegulatory activities documented in preclinical and clinical settings, indicating balanced, synergistic, and safe bioactivity.

**Methods:**

GB5’s phytochemical composition was standardized using gas chromatography–mass spectrometry (GC–MS) fingerprinting. In vitro assays assessed 2,2-diphenyl-1-picrylhydrazyl (DPPH) and nitric oxide (NO) scavenging capacity, inhibition of carbohydrate-digesting enzymes (α-amylase, α-glucosidase), enzyme kinetics, and effects on glucose uptake (GU) and lipid accumulation (LA) in yeast and 3T3-L1 adipocytes. Computational network pharmacology, molecular docking, and pharmacokinetic analyses elucidated molecular targets and bioavailability. Statistical analyses employed robust dose-response modelling, analysis of variance with Dunnett’s T3 test, and t-tests with false discovery rate correction.

**Results:**

GC–MS identified 21 bioactive compounds, including phytol, fatty acids, and sterols, driving GB5’s therapeutic synergy. GB5 showed robust antioxidant activity (DPPH and NO; half-maximal inhibitory concentration (IC_50_) 88.6 and 74.8 µg/mL) and mixed-type inhibition of α-amylase and α-glucosidase (IC_50_ 71.6 and 174 µg/mL). At sub-inhibitory doses, it outperformed ascorbic acid (ASA) and acarbose. In 3T3-L1 adipocytes, GB5 increased GU by 32.3% at 2.0 mg/mL, comparable to rosiglitazone, and reduced lipid accumulation by 18.6% (90% effective concentration (EC_90_) 0.742 mg/mL). Network pharmacology and molecular docking implicated peroxisome proliferator-activated receptor gamma (PPARγ), protein tyrosine phosphatase 1B (PTP1B), cyclooxygenase-2 (COX-2), and advanced glycation end-products–receptor for advanced glycation end-products (AGE–RAGE) pathways with 9,12-octadecadienoic acid and cholestan-3-ol, 2-methylene-, (3β,5α)- as key associates.

**Conclusions:**

GB5’s multi-targeted efficacy against hyperglycaemia, oxidative stress, and adipocyte dysfunction positions it as a promising complementary therapy for T2DM, meriting further in vivo evaluation.

**Graphical abstract:**

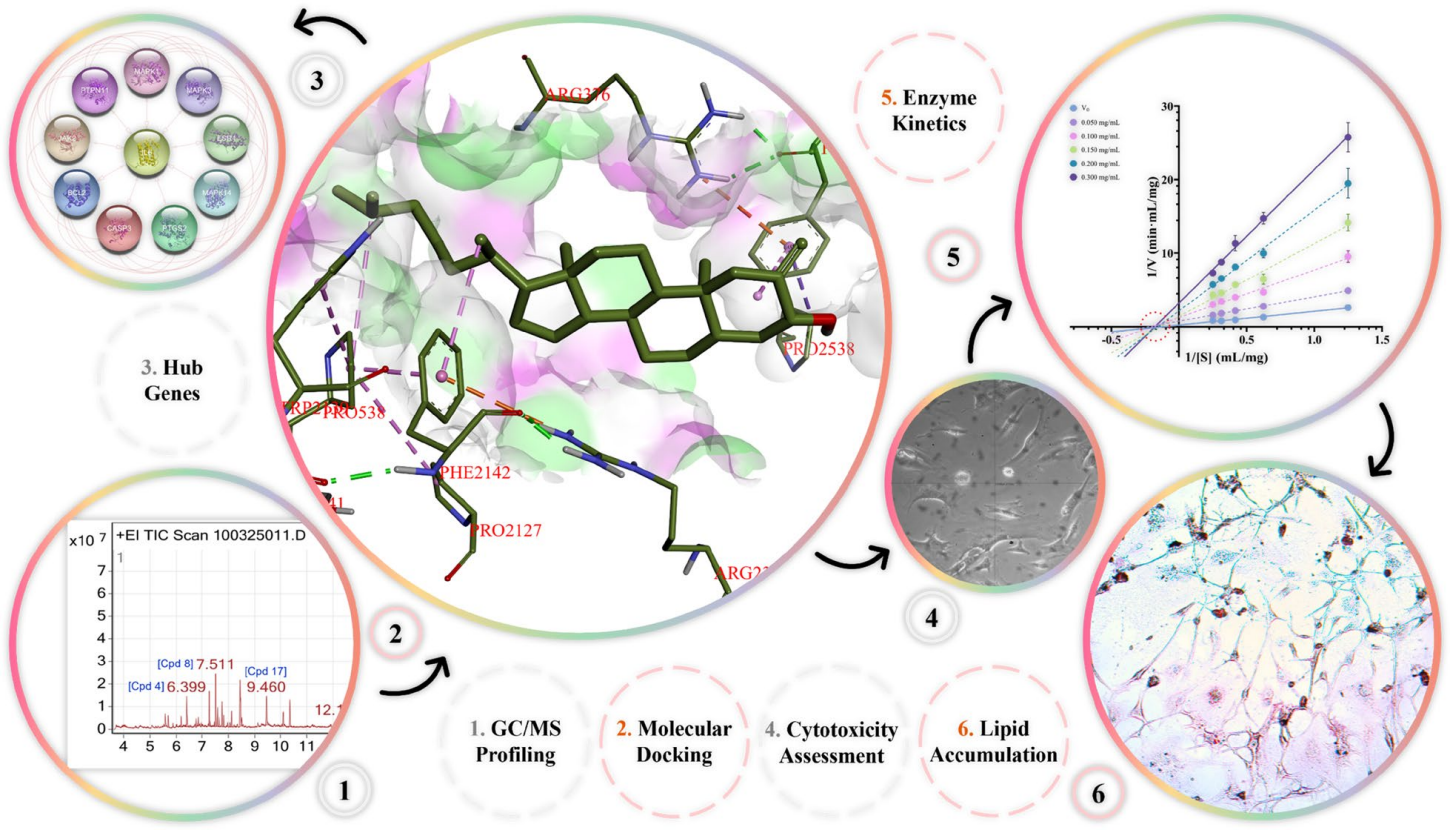

**Supplementary Information:**

The online version contains supplementary material available at 10.1186/s12906-025-05192-3.

## Introduction

Type 2 diabetes mellitus (T2DM) affects roughly 529 million people worldwide and may approach one billion by 2045. It drives most diabetes-related disability and more than 3 million deaths. Annual costs approach US$1 trillion, with the greatest burden in resource-limited settings [1]. Despite broad access to glucose-lowering agents, many patients progress from a prediabetic phase, characterized by modest biomarker elevations, to sustained hyperglycaemia (HbA_1_c ≥ 6.5%, fasting glucose (FG) ≥ 126 mg/dL) as compensatory hyperinsulinemia gives way to β-cell exhaustion [2]. Progression from prediabetes to sustained hyperglycaemia arises from ectopic lipid overload and inflammation driven by ER stress. Postprandial glucose (PPG) excursions, together with adipocyte cytokine signaling, blunt insulin action and impair mitochondrial function via stress-kinase pathways [1, 3].

Single-target agents lower glucose, but residual PPG, dyslipidaemia, and inflammatory tone often persist. Adverse effects can also limit adherence [4, 5]. Interest in complementary botanical therapies, especially polyherbal formulations (PHFs), is rising. Their widespread use underscores the need for standardized, mechanism-transparent products [6, 7]. Clinical surveys confirm robust patient interest and emerging efficacy data for glycaemic management [5, 7].

PHFs seek multi-target synergy, yet few are rigorously bio-standardized and mechanistic attribution is often limited. As adjuncts for patients insufficiently controlled by or intolerant to standard drugs, PHFs are widely used. Reports describe improvements in FG, PPG, and HbA_1_c, especially in early disease, along with favourable tolerability and accessibility [8–10]. Still, their heterogeneity and complexity argue for evidence-driven PHFs with quantitative biological standardization and transparent mechanisms [11, 12]. Accordingly, systematic preclinical evaluation with dose–response modelling is essential to define thresholds and feedback before clinical translation.

In this context, antidiabetic effects of individual botanicals are well documented, yet synergy in multi-herb formulations remains poorly defined. Reported advantages include enhanced efficacy and reduced doses of each component, minimizing adverse events. However, formulations with excessive botanicals complicate bio-standardization, obscure mechanisms of action, and impose logistical and economic burdens; many depend on traditional beliefs rather than rigorous science, limiting mechanistic clarity. To balance mechanistic clarity with therapeutic breadth, we formulated ‘GB5’ that integrates five botanicals with complementary mechanistic targets in T2DM pathophysiology, each supported by preclinical/clinical evidence as single agents or within PHFs. GB5 is a cold-percolated ethanolic extract, characterized by GC–MS fingerprinting to defined phytochemical ratios. Selection prioritized unique phytochemical signatures and underexploited volatile constituents. Detailed justification and literature support for each botanical, covering radical scavenging, carbohydrate-enzyme inhibition, insulin-sensitizing and lipid-modulating effects, and cardiometabolic protection, are provided in Table S1 in Supplementary Materials. Briefly, we combined selected parts of *Asparagus racemosus* Willd., *Cyperus rotundus* L., *Tinospora cordifolia* (Thunb.) Miers, *Terminalia arjuna* (Roxb.) Wight & Arn., and *Mimosa pudica* L. to target three axes relevant to early T2DM: (i) limiting postprandial carbohydrate hydrolysis; (ii) lowering inflammatory/oxidative tone; and (iii) modulating adipocyte lipid–glucose handling.

Supplementary table 1 (Table S1): Pharmacological attributes of selected botanicals individually and within PHFs supporting their inclusion in GB5.

The selection of these botanicals for GB5 is carefully crafted based on their individually validated pharmacological profiles and proven synergistic efficacy in PHFs, which align precisely with the metabolic improvements demonstrated in our study.

We aim to develop and preclinically validate GB5 as a multimodal candidate for early T2DM. Our approach was fourfold: (1) define the GC–MS fingerprint; (2) quantify antioxidant capacity and α-amylase/α-glucosidase inhibition with kinetics (IC_50_) and mode; (3) measure glucose uptake (GU) and lipid accumulation in 3T3-L1 adipocytes (EC metrics such as EC_50_); and (4) map putative pathways by network pharmacology, molecular docking, with in silico ADME/toxicity predictions. Dose–response relationships were modelled to anticipate in vivo behaviour. Here we show that GB5 inhibits α-amylase/α-glucosidase, attenuates oxidative stress, increases adipocyte GU, and modulates PI3K–AKT, AGE–RAGE, and PPAR signaling, supporting a mechanism-informed, tradition-derived polyherbal candidate for early T2DM.

## Experimental

### Materials

Key reagents included porcine pancreatic α-amylase; α-glucosidase (*S. cerevisiae*); soluble potato starch; p-NPG; PAHBAH; DNSA; DPPH; sodium nitroprusside; L-ascorbic acid; MTT; and Oil Red O. Acarbose and rosiglitazone served as standards. For cell assays, we used high-glucose DMEM, heat-inactivated FBS, and differentiation supplements (IBMX, dexamethasone, insulin). Full supplier, catalogue, and purity details are provided in Supplementary Materials.

### Preparation and extraction of plant material

For this study, roots and tubers of *A. racemosus* and *C. rotundus*, seeds of *T. cordifolia*, bark of *T. arjuna*, and leaves of *M. pudica* were collected in January 2024 from non-forest sites, fallow lands, and private properties in Tamil Nadu, India. Specifically, *A. racemosus* and *C. rotundus* were sourced from Thoothukudi district, and *T. cordifolia*, *T. arjuna*, and *M. pudica* from Tirunelveli district. Collections were conducted by an authorized commercial herbal dealer in Chengalpattu, Tamil Nadu, India. Authentication of specimens was performed by Dr. K. Jayeprakash, Institute of Natural Science Research, Tamil Nadu, and a voucher specimen was deposited under accession number INSciR/Herbarium/0089. The plant parts were shade-dried at ambient temperature (25–30 °C) for seven days, coarsely powdered, and mixed uniformly (200 g each, totalling 1 kg). The powdered mixture underwent extraction via cold percolation using ethanol (3 L) for 48 h. Subsequently, the extract was concentrated through rotary evaporation below 30 °C (PBV 7 d, Superfit Continental, India) and further lyophilized at 0 °C and 10 Pa (Samsung, Korea), yielding a final extract of 8.2 ± 0.3% w/w. We used cold ethanolic percolation to preserve thermolabile and volatile constituents, minimize thermal/oxidative artefacts, and maintain a composition suitable for downstream GC–MS analysis. Pre-defatting was not performed to retain semi-polar and lipophilic constituents. The extraction process was repeated thrice to ensure optimal yield. The resulting dried extract was stored at − 80 °C to minimize evaporative loss and oxidative degradation of volatile constituents prior to GC–MS and bioassays.

### Qualitative phytochemical screening of GB5

The polyherbal extract was subjected to qualitative phytochemical screening to identify the presence of various bioactive constituents. Established scientific methodologies were employed for all assessments. The phytochemical constituents evaluated included alkaloids, carbohydrates, glycosides, cardiac glycosides, proteins, amino acids, flavonoids, phenolic compounds, tannins, saponins, phytosterols, triterpenoids, quinones, anthraquinones, coumarins, and fixed oils and fats [13–16].

### GC-MS analysis

GC–MS fingerprinting was performed on a PerkinElmer Clarus 680 GC coupled to a Clarus 600 MS (EI). Lyophilized extract (2 g) was dissolved in ethanol (10 mL), passed through a 0.45-µm PTFE filter, dried over anhydrous Na_2_SO_4_, concentrated to 1 mL under N_2_ at 25 °C, and injected (1 µL; split 10:1). Separation used an Elite-5MS column (30 m × 0.25 mm × 0.25 μm) with helium (1.0 mL/min). Full-scan spectra (m/z 50–600) were acquired in TurboMass v5.4.2 and matched to the NIST 2008 library. Triplicate injections and solvent/matrix blanks verified retention-time (T_R_) and peak-area reproducibility and screened for artifacts. Full operating parameters (injector, oven program, MS source, acquisition settings) are provided in Supplementary Materials.

### Bioinformatics

#### ADMET analysis

An integrated in silico analysis assessed the ADMET profile and pharmacological relevance of identified compounds. Canonical SMILES and SDF files were sourced from PubChem (https://pubchem.ncbi.nlm.nih.gov/) and verified with NIST Chemistry WebBook (https://webbook.nist.gov/chemistry/). Pharmacokinetic insights were obtained via SwissADME (http://www.swissadme.ch/) bioavailability radars. Toxicity profiling utilized the Deep-PK Predictor (https://biosig.lab.uq.edu.au/deeppk/), a deep learning platform from the University of Queensland, employing convolutional neural networks trained on extensive human clinical datasets to predict parameters including AMES test results, micronucleus test outcomes, oral bioavailability, and intestinal absorption probabilities [17].

#### Network construction and analysis

SDF files for GB5 compounds were queried in SwissTargetPrediction (*Homo sapiens*); targets with prediction probability ≥ 10% were retained. Disease genes were retrieved from GeneCards (relevance score ≥ 10) using the keywords “Diabetes Mellitus” and “Lipid Metabolism”. Overlap with compound targets was analysed in STRING (*Homo sapiens*, evidence mode, minimum confidence 0.7) to build a high-confidence PPI network. Functional enrichment (GO Biological Process; Kyoto Encyclopaedia of Genes and Genomes (KEGG), Reactome, WikiPathways) was ranked by FDR, and the top 5–10 pathways were visualized in ChiPlot. Compound-target pathway networks were assembled in Cytoscape; hub genes were identified with CytoHubba (MCC), modules with MCODE (parameters in Supplementary Materials), topology verified via stringApp, and figures rendered with standard layout algorithms [18, 19].

### Molecular docking

Crystal structures were obtained from the PDB (https://www.rcsb.org/) for PPARγ LBD (6MD4), COX-2 (1CVU), PTP1B allosteric site (1T49), and the RAGE VC1 domain (6XQ3). Where available, the co-crystallized ligand defined the binding pocket. Receptors were prepared in PyMOL 2.0: distant waters/ions were removed; essential cofactors and waters within ~ 3.5 Å of the ligand were retained; polar hydrogens were added; and pH-7.4 states for titratable residues were optimized. For PPARγ (6MD4), two receptor states reflecting the dual co-bound ligands were generated for validation. Structures were converted to PDBQT with Gasteiger charges.

Based on network pharmacological relevance, GB5 constituents (C3, C6, C7, C12, and C19) and reference controls (rosiglitazone or oleate for PPARγ; arachidonic acid for COX-2; 3-(3,5-dibromo-4-hydroxy-benzoyl)−2-ethyl-benzofuran-6-sulfonic acid (4-Sulfamoyl-Phenyl)-Amide (PubChem ID: 448661) for PTP1B; 3-(3-{[3-(4-carboxyphenoxy)phenyl]methoxy}phenyl)−1 H-indole-2-carboxylic acid (PubChem ID: 155937458) for RAGE) were built from PubChem SMILES. Relevant tautomers/stereoisomers were enumerated, protonation was set for pH ~ 7.4 (fatty acids as carboxylates), and geometries were minimized in Discovery Studio 4.5.

Docking used AutoDock Vina (via PyRx v0.9.x) with exhaustiveness = 32, num_modes = 20, energy_range = 3, and a fixed random seed. Grid boxes were centred on the co-crystal centroid and extended to enclose each pocket with a ~ 6–8 Å margin (PPARγ LBD, COX-2 cyclooxygenase channel, PTP1B allosteric cavity, RAGE VC1 site). Validation comprised redocking each co-crystallized ligand; runs with heavy-atom RMSD ≤ 2.0 Å versus the crystal pose were accepted, otherwise the grid/sampling was tuned. Test ligands were docked to rigid receptors, poses were clustered at 2.0 Å, and the top-scoring pose from the best cluster was analysed. Interactions annotated H-bonds (≤ 3.5 Å), salt bridges, π–π/π–cation, and hydrophobics to confirm plausibility. Vina affinities (kcal/mol) were used only for qualitative rank-ordering and compared to standards descriptively (better/similar/worse) [20].

### In vitro radical scavenging and enzymatic Inhibition assays

#### DPPH radical scavenging assay

DPPH radical scavenging activity was determined to assess the free radical quenching capacity of test extracts following a published method, with minor adjustments [21]. A 0.5 mM DPPH solution in methanol was freshly prepared and mixed with 1.0 mL of test extract at concentrations ranging from 1000 to 10 µg/mL, achieved via √log dilution (~ 3.16-fold reductions) in triplicate. To maintain linear response windows and stable curve fits with a complex, multi-component extract, we used higher reagent concentrations in select assays (e.g., DPPH 0.5 mM). This preserved initial-rate kinetics, improved signal-to-background, and avoided ceiling/floor effects that bias IC_50_ estimates. Vehicle content was kept low; solutions were clear; matrix/vehicle blanks and wavelength-matched background corrections were applied. Ascorbic acid (ASA) served as the reference standard, with methanol as the blank. Mixtures were incubated in the dark at room temperature (RT) for 30 min, and absorbance was measured at 517 nm using a Synergy HT Multi-Mode microplate reader. Scavenging percentage was calculated as:

1 $$\:Scavenging\:\left(\%\right)=\left(\frac{{OD}_{Control}-{OD}_{Sample}}{{OD}_{Control}}\right)\times\:100$$

#### Nitric oxide scavenging assay

Nitric oxide (NO) scavenging activity was measured using 10 mM sodium nitroprusside in PBS (pH 7.4) as the NO source. A 2.0 mL nitroprusside solution was combined with 0.5 mL of test extract or ASA at concentrations matching the DPPH assay, prepared in triplicate. Mixtures were incubated at RT under light for 150 min to generate NO. Subsequently, 0.5 mL of Griess reagent, comprising equal volumes of 1% sulphanilamide in 2% phosphoric acid and 0.1% N-(1-naphthyl)-ethylenediamine dihydrochloride, was added, followed by 30 min incubation for diazotization [21]. Absorbance at 547 nm determined NO inhibition, calculated using Eq. 1.

#### α-Amylase inhibitory activity

Porcine pancreatic α-amylase (2 U/mL) was dissolved in ice-cold distilled water, and 3% (w/v) potato starch was prepared in 20 mM PBS (pH 6.9) by boiling until clear. Test samples and acarbose, at concentrations matching the DPPH assay, were mixed with 80 µL of enzyme solution and incubated at RT for 10 min in triplicate. After adding 40 µL of starch solution and incubating for another 10 min, the reaction was stopped with 80 µL of DNSA and heated at 95 °C for 5 min [22]. Absorbance at 540 nm determined inhibition, calculated as:

2 $$\:Inhibition\:\left(\%\right)=\left(\frac{{A}_{C}-{A}_{CB}-({A}_{E}-{A}_{SB})}{{A}_{C}-{A}_{CB}}\right)\times\:100$$

Where ‘*A*_*C*_’ is control reaction absorbance with active enzyme and PBS, ‘*A*_*CB*_’ is control blank with denatured enzyme and PBS, ‘*A*_*E*_’ is experimental extract with active enzyme and inhibitor, and ‘*A*_*SB*_’ is sample blank with denatured enzyme and inhibitor. Substrate was included in all setups.

#### α-Glucosidase inhibitory activity

α-Glucosidase (0.4 U/mL) from *Saccharomyces cerevisiae* was dissolved in 0.1 M PBS (pH 6.8) with 0.2% bovine serum albumin. Test samples and acarbose, at concentrations matching the α-amylase assay, were mixed with 20 µL of enzyme solution and 20 µL of 12 mM p-nitrophenyl-α-D-glucopyranoside substrate in triplicate. After 15 min incubation at 37 °C, 80 µL of 0.2 M sodium carbonate stopped the reaction [22]. Absorbance at 405 nm determined inhibition, calculated using Eq. 2.

#### Enzyme inhibition kinetics

Kinetic parameters for GB5 against α-amylase and α-glucosidase were determined using 1.0 mL reaction mixtures with substrate concentrations of 0.8–4.0 mg/mL potato starch for α-amylase (100 mM PBS, pH 7.0) and 5–25 mM p-nitrophenyl-α-D-glucopyranoside for α-glucosidase (100 mM PBS, pH 6.8). GB5 concentrations ranged from 0 to 0.300 mg/mL for α-amylase and 0–0.800 mg/mL for α-glucosidase. Enzyme concentrations (0.4–1.6 U/mL for α-amylase; 0.9–2.7 U/mL for α-glucosidase) ensured linear product formation. Reactions were initiated by enzyme addition, and 300 µL aliquots were sampled at 2, 5, 8, 12, and 15 min. For α-amylase, aliquots were quenched with 1.2 mL of 0.3 M sodium carbonate, and reducing sugar was quantified using the PAHBAH method. For α-glucosidase, aliquots were quenched with 200 µL of 0.1 M sodium carbonate, and absorbance was measured at 405 nm. Initial rates from linear product-time plots (R^2^ ≥ 0.99) were fitted to a mixed-inhibition model. Reciprocal plots (1/v vs. 1/[S]) assessed mixed-inhibition, extracting competitive (K_ic_) and uncompetitive (K_iu_) constants. Dixon plots (1/v vs. [I]) validated these findings [23, 24].

### Analysis of GU using yeast cells

GU enhancement by test extracts was assessed using a *Saccharomyces cerevisiae* model [25]. A 1% w/v baker’s yeast suspension in distilled water was incubated overnight at RT, centrifuged at 4200 rpm for 5 min (Centrifuge 5425 R, Eppendorf, Germany) until a clear supernatant was obtained, and diluted to a 10% v/v working suspension. Assay mixtures combined 1 mL of test sample in 5 mM glucose solution at concentrations from 2 to 0.125 mg/mL via log_2_ dilutions in triplicate, with control tubes containing glucose alone. After 10 min incubation at 37 °C, 100 µL of yeast suspension was added, mixed, and incubated for 60 min. Tubes were centrifuged at 3800 rpm for 5 min. Metronidazole at 200 µM served as the reference standard. Supernatant glucose content was quantified using the GOD–POD method at 520 nm with a semi-automatic biochemical analyser (BTS-350, BioSystems). GU percentage was calculated using Eq. 1.

### Maintenance of 3T3-L1 preadipocytes

3T3-L1 mouse embryonic fibroblasts, sourced from the National Centre for Cell Science, were cultured in DMEM with high glucose, L-glutamine, 10% FBS, and 1% penicillin/streptomycin in T25 flasks (TARSONS, Korea) at 37 °C with 5% CO_2_ in a humidified incubator (Steri-Cycle i160, Thermo Scientific). Cells were passaged at 70–80% confluency in a 1:3 ratio using 0.25% trypsin-EDTA after washing with DPBS. Viability, assessed via trypan blue exclusion with a haemocytometer (Hausser Scientific, USA), exceeded 95%. Culture medium was renewed every 2–3 days.

### Assessment of cytotoxicity using MTT

3T3-L1 preadipocytes were seeded at 2 × 10^4^ cells/well in 24-well plates (Corning, USA). Test extracts at 2 mg/mL and four lower concentrations via log_2_ serial dilution were evaluated in triplicate. After overnight attachment, cells were exposed to test items for 48 h. Triton X-100 at 0.1% v/v served as the cytotoxic standard. MTT solution at 5 mg/mL was added, incubated for 4 h at 37 °C, and formazan crystals were dissolved in DMSO. Absorbance at 550 nm determined viability as follows:

3 $$\:Viability\:\left(\%\right)=\left(\frac{{OD}_{Exposed\:Cells}-{OD}_{Blank\:Control}}{{OD}_{Vehicle\:Control}-{OD}_{Blank\:Control}}\right)\times\:100$$

Cytotoxicity was defined as a 20% growth reduction compared to vehicle control, with morphological changes graded from 0 to 4 (4 being severely cytotoxic) under an inverted microscope (AE31, Motic).

### Differentiation of 3T3-L1 fibroblasts to mature adipocytes

Adipogenic differentiation followed a modified protocol [26]. 3T3-L1 fibroblasts were seeded at 4 × 10^4^ cells/well in 24-well plates in DMEM with 10% FBS and 1% penicillin/streptomycin. At 70–80% confluency, differentiation was induced with medium A (DMEM with 0.25 mM dexamethasone, 0.5 mM IBMX, 1 µM insulin, 4 mM L-glutamine, 10% FBS, 1% penicillin/streptomycin) for 72 h. Medium B (DMEM with 1 µM insulin, 4 mM L-glutamine, 10% FBS, 1% penicillin/streptomycin) was applied for 48 h, followed by standard DMEM for 72–120 h with medium renewal every 48 h. Morphological changes and lipid droplet accumulation confirmed adipogenesis.

### Analysis of lipid accumulation in matured adipocytes

Lipid accumulation (LA) in differentiated 3T3-L1 adipocytes was assessed using Oil Red O staining [27]. Test extracts at concentrations from 2 to 0.125 mg/mL were applied from day 4 post-differentiation until maturation. Cells were fixed with 10% neutral buffered formalin for 1 h, rinsed with PBS, and incubated with 60% isopropanol. After drying, 0.3% w/v Oil Red O solution in 60% isopropanol was applied for 1 h. Excess dye was removed with PBS, and images were captured using a trinocular microscope (B-600 TiFL, OPTIKA). Lipid-bound dye was eluted with 100% isopropanol, and absorbance at 550 nm was quantified. Rosiglitazone at 5 µM served as the reference standard. Total LA was calculated:

4 $$\:{TLA}_{\%Diff.}=\left(\frac{{OD}_{Treated\:Cells}-{OD}_{Negative\:Control}}{{OD}_{Negative\:Control}}\right)\times\:100$$

### Analysis of GU in mature adipocytes

GU in differentiated 3T3-L1 adipocytes was evaluated [27]. Preadipocytes, seeded at 4 × 10^4^ cells/well in 24-well plates, underwent differentiation. Mature adipocytes were serum-starved for 2 h in serum-free DMEM, then treated with extracts at 0.20, 0.63, and 2.0 mg/mL in glucose-free DMEM for 5 h at 37 °C with 5% CO_2_. A 5 µL sample was mixed with 500 µL of GOD-POD reagent, incubated for 20 min, and absorbance was measured at 500 nm. Rosiglitazone at 5 µM served as the reference standard. GU was calculated:

5 $$\:{GU}_{\%Diff.}=\left(\frac{{OD}_{Negative\:Control}-{OD}_{Treated\:Cells}}{{OD}_{Negative\:Control}}\right)\times\:100$$

### Statistical analysis

Analyses were performed in GraphPad Prism 8.0.2 (build 263) and Microsoft Excel 2021. Normality was tested with Shapiro–Wilk (α = 0.05). Concentration–response data for antioxidant, enzyme-inhibition, and MTT assays were fit to a 4PL model with log_10_ concentration on the x-axis; IC_50_/EC values are reported with 95% CIs. Model adequacy was evaluated by extra sum-of-squares F tests.

Enzyme-kinetic parameters (K_m_, V_max_, K_ic_, K_iu_) were derived from Lineweaver–Burk and Dixon plots using linear regression of initial rates. For GU assays, EC_50_ in yeast was estimated by cubic polynomial regression; 3T3-L1 adipocyte uptake used linear regression. Lipid accumulation was modelled with a one-phase exponential decay. Outliers were screened by Dixon’s Q (1%).

Group comparisons used multiple unpaired t-tests with Welch’s correction and FDR control at 0.05 (Benjamini–Krieger–Yekutieli) for antioxidant/enzyme assays. For yeast/adipocyte GU and LA, we applied one-way ANOVA with Brown–Forsythe/Welch adjustments and Dunnett’s T3 post hoc tests. All tests were two-sided with *p* < 0.05 considered significant. Sample sizes (n), replicate structure, and exact comparisons are detailed in figure legends. Full equations, constraints, and diagnostics are provided in Methods S1 (Supplementary).

## Results

### Qualitative phytochemical analysis of GB5

Qualitative phytochemical analysis of the PHF, composed of five selected botanicals, identified a diverse profile of bioactive constituents. The extract exhibited strong positive reactions for alkaloids, proteins/amino acids, and tannins, with moderate concentrations of phenolic compounds, flavonoids, and phytosterols (Table S2 in Supplementary Materials). Glycosides, saponins, and triterpenoids were minimally detected or absent.

Supplementary table 2 (Table S2): Qualitative phytochemical analysis of GB5.

The table presents results from standardized tests on the polyherbal extract, indicating the presence (+, ++, +++) or absence (–) of major phytochemical classes. This profile summarizes the bioactive constituents contributed by the five constituent botanicals.

### Chemical profiling of GB5 by GC-MS

**Fig. 1:**
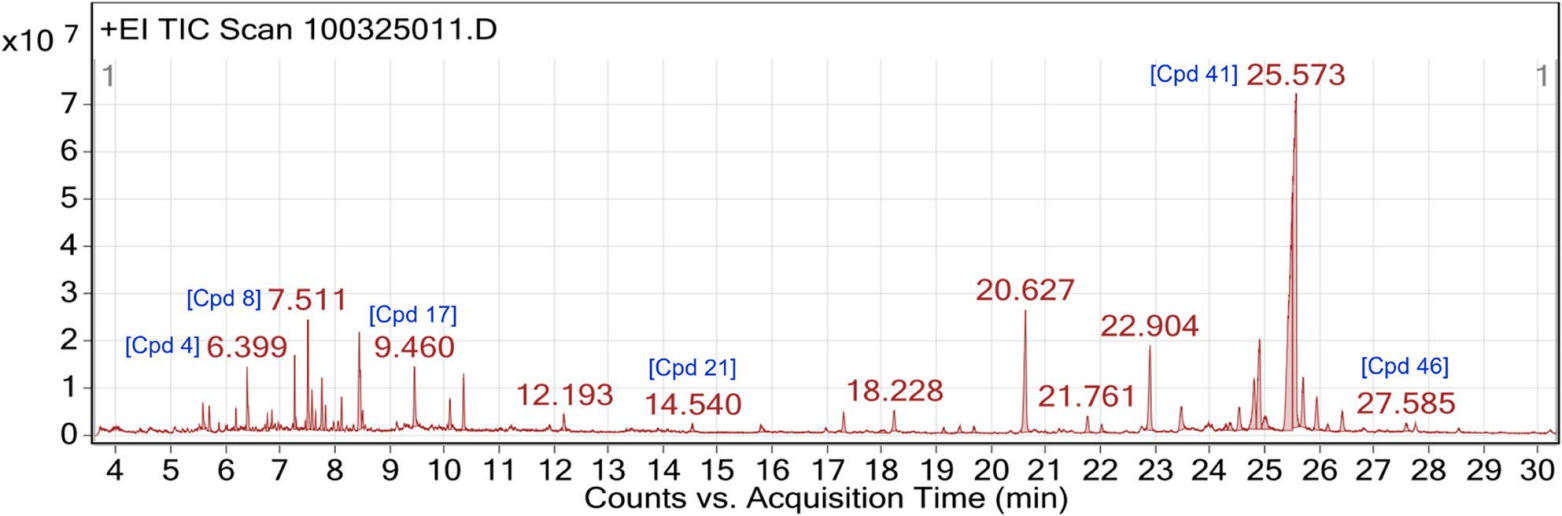
GC–MS total-ion chromatogram (TIC) of GB5 (4–30 min). Major peaks represent putatively annotated constituents listed in Table 1; peak intensity reflects relative abundance. The stable baseline indicates adequate separation and low background

**Fig. 2:**
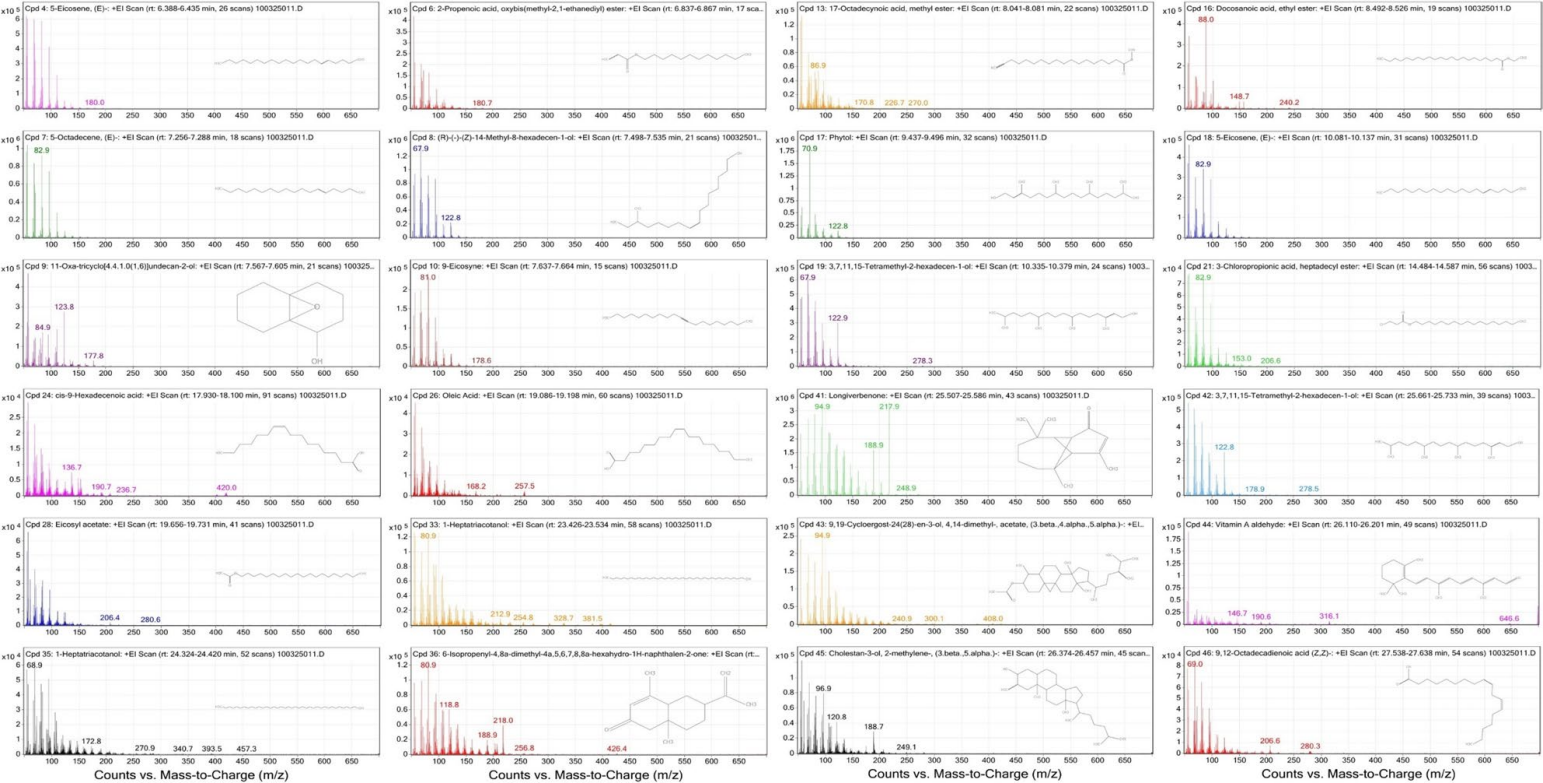
Mass spectra of major GC–MS-identified constituents in GB5. Each panel corresponds to compounds C1–C21, identified by system-assigned (Cpd) numbers matching user-assigned numbers in Table 1. The main plot displays ion counts versus mass-to-charge ratio (m/z), with dominant fragment peaks annotated. An inset TIC trace illustrates the elution profile at the compound’s T_R_. Structural diagrams, based on NIST library matches, are included when available

GC–MS analysis of the ethanolic extract of the PHF revealed a diverse chemical profile suggestive of phytotherapeutic synergy. The total-ion-current chromatogram, electron-impact mass spectra of major compounds, and chromatographic details are presented in Figs. 1 and 2, and Table 1, respectively. The analysis tentatively annotated 21 phytoconstituents by EI–MS NIST library matching and T_R_s ranging from 6.4 to 27.6 min. These compounds included long-chain fatty acids and esters (e.g., C6, C7), diterpenoid alcohols (e.g., C4, C5), and sterol-like constituents.

**Table 1:** Chemical composition of GB5 by GC–MS

ACN	Cpd	Compound label	DB formula	T_*R*_ (min)	Height	Height%	Area	Area %	Area Sum %	Symmetry	Width
C1	4	5-Eicosene, (E)-	C_20_H_40_	6.399	13339620.14	18.94	19316388.46	6.9	1.68	3.1	0.087
C1	18	5-Eicosene, (E)-	C_20_H_40_	10.109	6334363.17	8.99	10989512.29	3.92	0.96	1.16	0.062
C2	7	5-Octadecene, (E)-	C_18_H_36_	7.269	15623768.54	22.18	16243112.02	5.8	1.42	1.43	0.04
C3	16	Docosanoic acid, ethyl ester	C_24_H_48_O_2_	8.509	3540896.94	5.03	4544832.32	1.62	0.4	1.06	0.041
C4	17	Phytol	C_20_H_40_O	9.46	12517454.23	17.77	24764707.73	8.84	2.16	1.57	0.105
C5	19	3,7,11,15-Tetramethyl-2-hexadecen-1-ol	C_20_H_40_O	10.358	11819716.36	16.78	19319209.42	6.9	1.68	0.9	0.1
C5	42	3,7,11,15-Tetramethyl-2-hexadecen-1-ol	C_20_H_40_O	25.699	10551158.58	14.98	26704133.17	9.53	2.33	0.84	0.108
C6	24	cis-9-Hexadecenoic acid	C_16_H_30_O2	18.026	604746.27	0.86	3,693,201	1.32	0.32	0.56	0.181
C7	26	Oleic Acid	C_18_H_34_O_2_	19.132	1221071.48	1.73	3174257.95	1.13	0.28	1.33	0.128
C8	43	9,19-Cycloergost-24(28)-en-3- ol, 4,14-dimethyl-, acetate, (3β,4α,5α)-	C_32_H_52_O_2_	25.954	6951779.63	9.87	19380711.03	6.92	1.69	0.67	0.112
C9	28	Eicosyl acetate	C_22_H_44_O_2_	19.682	1328277.43	1.89	3803668.99	1.1	0.27	1.32	0.098
C10	33	1-Heptatriacotanol	C_37_H_76_O	23.472	4902324.4	6.96	16549278.79	5.91	1.44	1.67	0.147
C10	35	1-Heptatriacotanol	C_37_H_76_O	24.366	1635814.61	2.32	5061363.04	1.81	0.44	1.1	0.109
C11	41	Longiverbenone	C_15_H_22_O	25.573	70448698.29	100	280,072,007	100	24.43	0.21	0.123
C12	46	9,12-Octadecadienoic acid (Z, Z)-	C_18_H_32_O_2_	27.585	1692665.47	2.4	5548318.39	1.98	0.48	1.02	0.131
C13	6	2-Propenoic acid, oxybis(methyl-2,1-ethanediyl) ester	C_15_H_28_O_2_	6.852	3991534.53	5.67	3871122.81	1.38	0.34	1.07	0.036
C14	8	(R)-(-)-(Z)−14-Methyl-8-hexadecen-1-ol	C_17_H_34_O	7.511	23153684.78	32.87	27546297.15	9.84	2.4	1.68	0.061
C15	9	11-Oxatricyclo[4.4.1.0(1,6)]undecan	C_10_H_16_O_2_	7.584	8271474.04	11.74	10142502.38	3.62	0.88	1.21	0.061
C16	13	17-Octadecynoic acid, methyl ester	C_19_H_34_O_2_	8.064	1906331.66	2.71	2813379.24	1	0.25	0.8	0.055
C17	36	6-Isopropenyl-4,8a-dimethyl-4a,5,6,7,8,8a-hexahydro-1 H-naphthalen-2-one	C_15_H_22_O	24.526	4844780.88	6.88	14821882.94	5.29	1.29	1.52	0.145
C18	44	Vitamin A aldehyde	C_20_H_28_O	26.152	1482976.69	2.11	3879365.7	1.39	0.34	0.87	0.102
C19	45	Cholestan-3-ol, 2-methylene-, (3β,5α)-	C_28_H_48_O	26.414	4165870.38	5.91	11567851.46	4.13	1.01	1.13	0.133
C20	21	3-Chloropropionic acid, heptadecyl ester	C_20_H_39_ClO_2_	14.54	1519455.47	2.16	3479653.85	1.24	0.3	1.06	0.123
C21	10	9-Eicosyne	C_20_H_38_	7.65	3810834.28	5.41	3716541.05	1.33	0.32	0.96	0.041

The table lists compounds C1–C21 in order of increasing T_R_, including tentative identities, molecular formulas, etc. System-assigned (Cpd) and user-assigned (ACN) compound numbers are provided. Peak height, relative area (%), and area sum (%) quantify compound abundance and contribution to total signal. Peak symmetry and width describe chromatographic resolution. The profile encompasses low-boiling aliphatics, medium-polarity alcohols/esters, and high-boiling lipids and sterols, reflecting the extract’s chemical diversity

Quantitative analysis of integrated chromatographic data indicated that most bioactive peaks had area percentages ranging from 1% to 20%, with a median of 5% to 10%. However, the distribution was skewed, with a subset of late-eluting peaks (T_R_ >20 min) contributing disproportionately to the total chromatographic area. Mass spectral analysis of the 21 curated compounds provided robust diagnostic evidence. Characteristic fragment ions, including [M–CH_3_]⁺ and [M–H_2_O]⁺ losses, matched reference spectra, supporting the assigned structures. For example, the mass spectrum of phytol (C4, T_R_ 9.46 min) exhibited fragmentations consistent with its diterpenoid structure.

Six compounds (Cpd 4, 8, 17, 21, 41, 46) were directly annotated on the GC–MS chromatogram, supported by strong spectral matches and high peak resolution. The remaining 15 compounds were identified via a user-generated chromatogram peak list based on consistent T_R_s, match factors, and integration integrity. These included compounds associated with sterol and retinoid pathways (e.g., C9, C18, C19), and less common lipid-like structures (e.g., C16, C17, C20), potentially modulating membrane integrity or oxidative stress [28, 29]. Some compounds appeared at multiple T_R_s, likely due to isomeric variation, matrix-induced shifts, or partial thermal modification. These instances were consolidated to maintain a count of unique constituents.

### Mechanistic insights into phytocompound activity via systems pharmacology

**Fig. 3:**
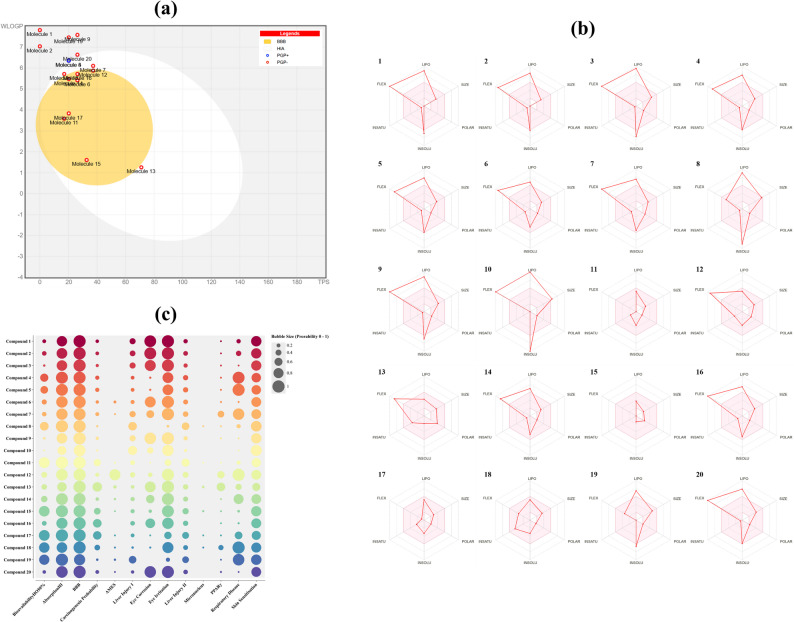
Pharmacological profiling of GB5 phytoconstituents. **a** TPSA vs WLOGP (BOILED-Egg) for compounds C1–C20 (corresponding to ACN 1–20 in Table 1): white zone, high human intestinal absorption; yellow zone, likely BBB permeation. Points: blue, P-gp substrates (PGP^+^); red, non-substrates (PGP^–^). **b** Bioavailability radar summarizing lipophilicity, size, polarity, solubility, saturation, and flexibility. **c** ADMET bubble map across endpoints; bubble size encodes predicted probability (0–1); absent bubbles indicate values below the display threshold

To investigate the mechanistic roles of GC–MS-identified phytocompounds, a systems pharmacology workflow was conducted. C21/Cpd 10 (9-eicosyne) was excluded due to the absence of targets meeting selection criteria. The remaining 20 compounds underwent ADMET profiling using SwissADME and Deep-PK, with results visualized in a TPSA versus WLOGP plot, spider plots, and a bubble plot (Fig. 3a, b and c, respectively). These analyses revealed a heterogeneous but favourable pharmacokinetic profile, with most compounds demonstrating high predicted human intestinal absorption, moderate blood-brain barrier permeability, and low toxicity indices, while maintaining lipophilicity and solubility within drug-likeness boundaries (Fig. 3a and b). The bubble plot highlighted the ADMET and safety landscape, with bubble size indicating endpoint probability (Fig. 3c). C4, C5, C8, C11, C15, and C17–C19 exhibited high oral bioavailability, strong intestinal absorption, and notable blood-brain barrier permeation, suggesting potential central activity. Mutagenicity (AMES), carcinogenicity, and micronucleus formation probabilities were consistently low, indicating a benign safety profile, while eye corrosion, irritation, and skin sensitization probabilities aligned with expectations for volatile phytochemicals.

**Fig. 4:**
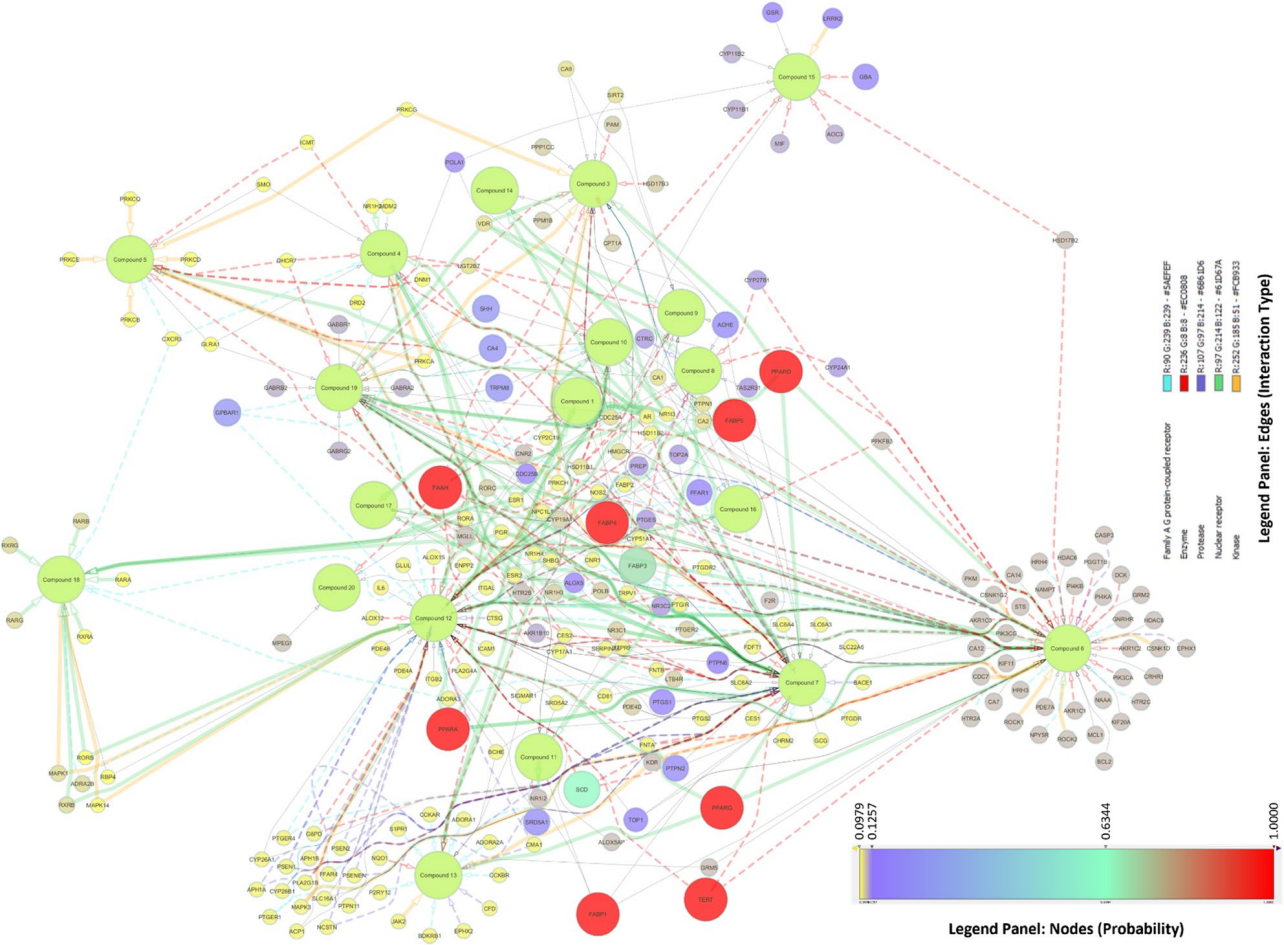
Predicted interactions of polyherbal phytoconstituents with human targets. GC–MS–annotated constituents (green nodes, C1–C20; Table 1) connect to predicted targets. Target nodes are coloured by prediction probability (blue → red, low → high). Node size = degree; edge width = interaction score; edge colours denote target classes (enzymes, GPCRs, ion channels, kinases, nuclear receptors, other). C7 and C12 show high connectivity to PPARs/FABPs/FAAH

**Fig. 5:**
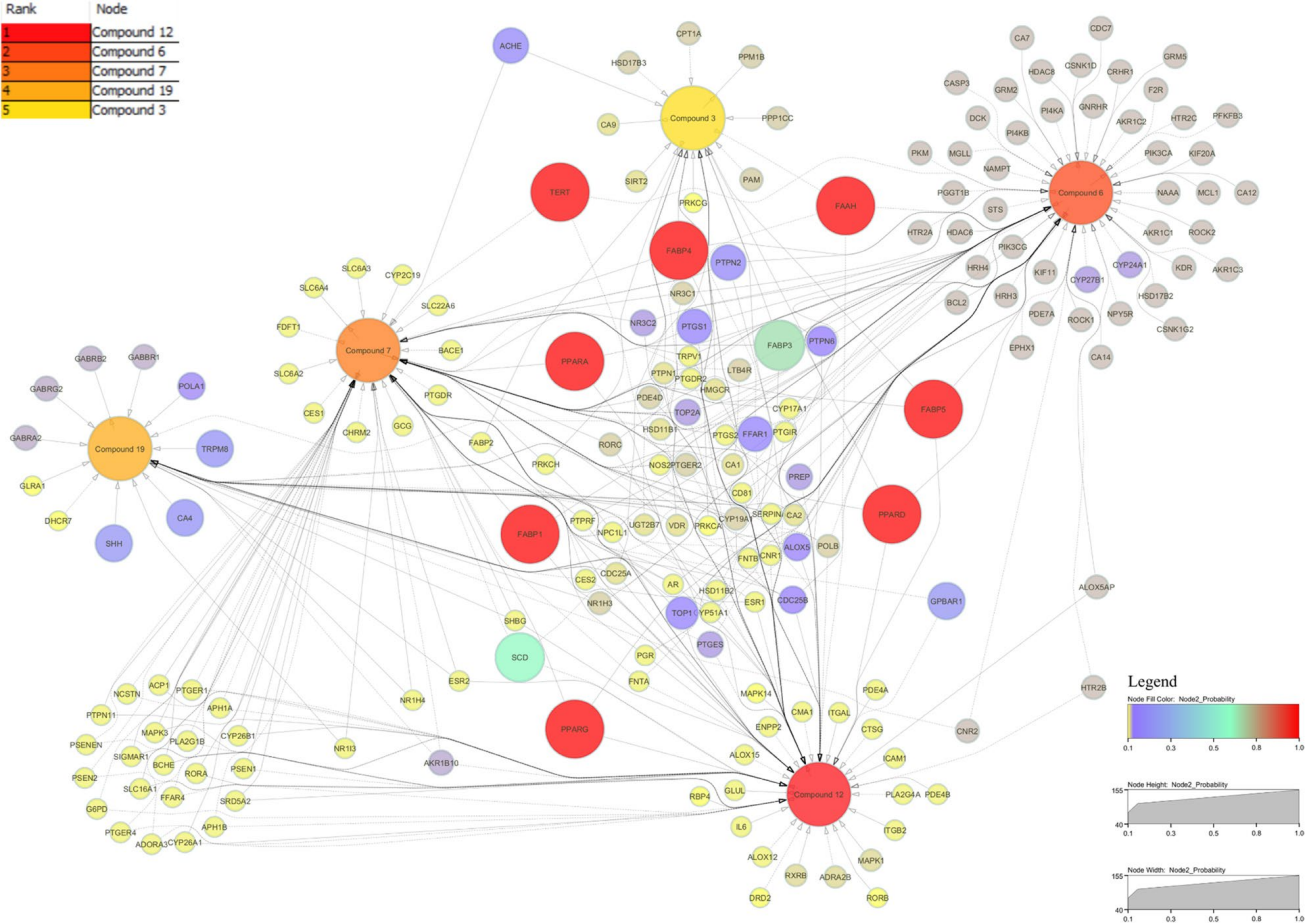
Topological ranking of compound-target interactions. The network, analysed using the CytoHubba MCC algorithm in Cytoscape, ranks compounds by topological centrality. Node colour (deep yellow to red) and size encode MCC scores, with red indicating the highest centrality. C3, C6, C7, C12, and C19 are the top five hubs, engaging multiple high-confidence targets in metabolic and inflammatory pathways. Solid edges depict all predicted interactions; dashed edges highlight the top 30% most abundant links

For target identification, the 20 compounds were analysed using SwissTargetPrediction, filtered for human-specific proteins with a prediction probability ≥ 10%. The compound-target interactions, including interaction types, target classes, and probabilities, are presented in Fig. 4. The matrix was imported into Cytoscape v3.10.3. Using CytoHubba with the MCC algorithm, C12 ranked highest with a score of 102, followed by C6 with 101, C7 with 93, C19 with 36, and C3 with 27. Each connected to multiple nodes governing metabolism, inflammation, and apoptosis (Fig. 5). MCODE on the compound–target network, with degree cutoff 10, node density at least 0.5, node score at least 0.5, K-core 2, and maximum depth 100, resolved two dense modules. Cluster 1 scored 2.0 with 11 nodes and 12 edges and centred on the protein kinase C family, PRKCB, PRKCE, PRKCQ, PRKCD, together with MDM2, CDC25A, AR, NR1H2, and C4, C5, and C10, indicating a PKC-driven hub that modulates cell-cycle control, apoptosis, and nuclear-receptor transcription. Cluster 2 scored 1.6 with 4 nodes and 4 edges and linked SHBG and CYP19A1 with C11 and C17, highlighting a steroid-metabolism module and hormone-responsive pathways. These clusters suggest that the PHF’s phytocompounds target kinase-mediated signal transduction and hormone-modulating pathways, supporting their anti-inflammatory and metabolic regulatory potential.

**Fig. 6:**
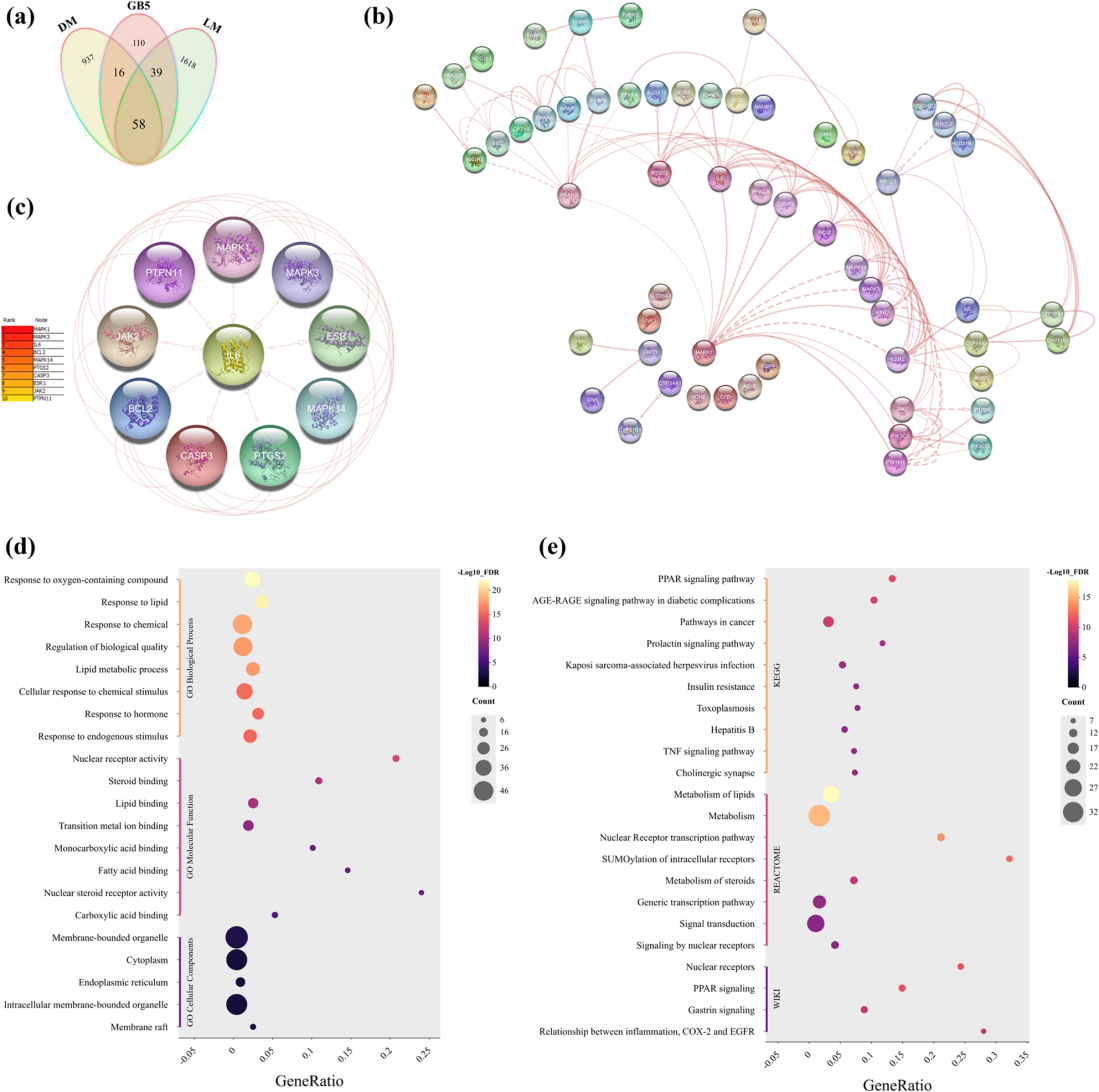
Network and enrichment analysis of polyherbal target genes. **a** Venn diagram showing 57 genes common to compound targets, diabetes genes, and lipid-metabolism genes. **b** STRING PPI network (confidence ≥ 0.70); MAPK1/MAPK3/IL-6/BCL2/MAPK14 emerge as central hubs. **c** Top-10 hub genes ranked by MCC. **d–e** Enrichment plots for the overlap genes: (**d**) KEGG pathways; (**e**) GO Biological Process (FDR < 0.05). Bubble size = gene count; colour = −log10(FDR); x-axis = gene ratio

Disease relevance was assessed by intersecting compound-associated genes with protein-coding genes annotated for “Diabetes Mellitus” and “Lipid Metabolism” in GeneCards (relevance score ≥ 10). This analysis identified 57 overlapping targets (Fig. 6a), likely representing key modulatory nodes. These targets were analysed in STRING (*Homo sapiens*, experimentally validated/database-derived interactions, confidence score ≥ 0.7, edge meaning set to “evidence”), generating a protein-protein interaction network (Fig. 6b). Using stringApp v2.2.0 in Cytoscape, CytoHubba ranked MAPK1 score 940, MAPK3 924, IL-6 908, BCL2 866, and MAPK14 with 864 as top hubs, followed by PTGS2, CASP3, ESR1, JAK2, and PTPN11 (Fig. 6c). STRING network enrichment at FDR ≤ 0.05 highlighted the GO terms: response to lipid GO:0033993; lipid metabolic process GO:0006629; lipid binding GO:0008289; and nuclear receptor activity GO:0004879 (Fig. 6d). Pathway analysis across KEGG, WikiPathways, and Reactome identified PPAR signaling, MAPK cascade, lipid metabolism, and AGE–RAGE signaling in diabetic complications as dominant pathways (Fig. 6e), visualized using ChiPlot. Network layouts were refined with yFiles Layout Algorithms v1.1.5 and annotated with Legend Creator v1.1.7 for clarity.

Integration of compound, target, and pathway data positioned IL-6 as a central mediator, interacting with MAPK1, MAPK3, MAPK14, PTGS2, and BCL2, genes associated with inflammation-induced insulin resistance and adipocyte dysfunction. This interconnected network supports the formulation’s multi-target synergy and provides a mechanistic basis for its observed antidiabetic activity [30, 31].

### Insights from molecular docking analysis

**Fig. 7:**
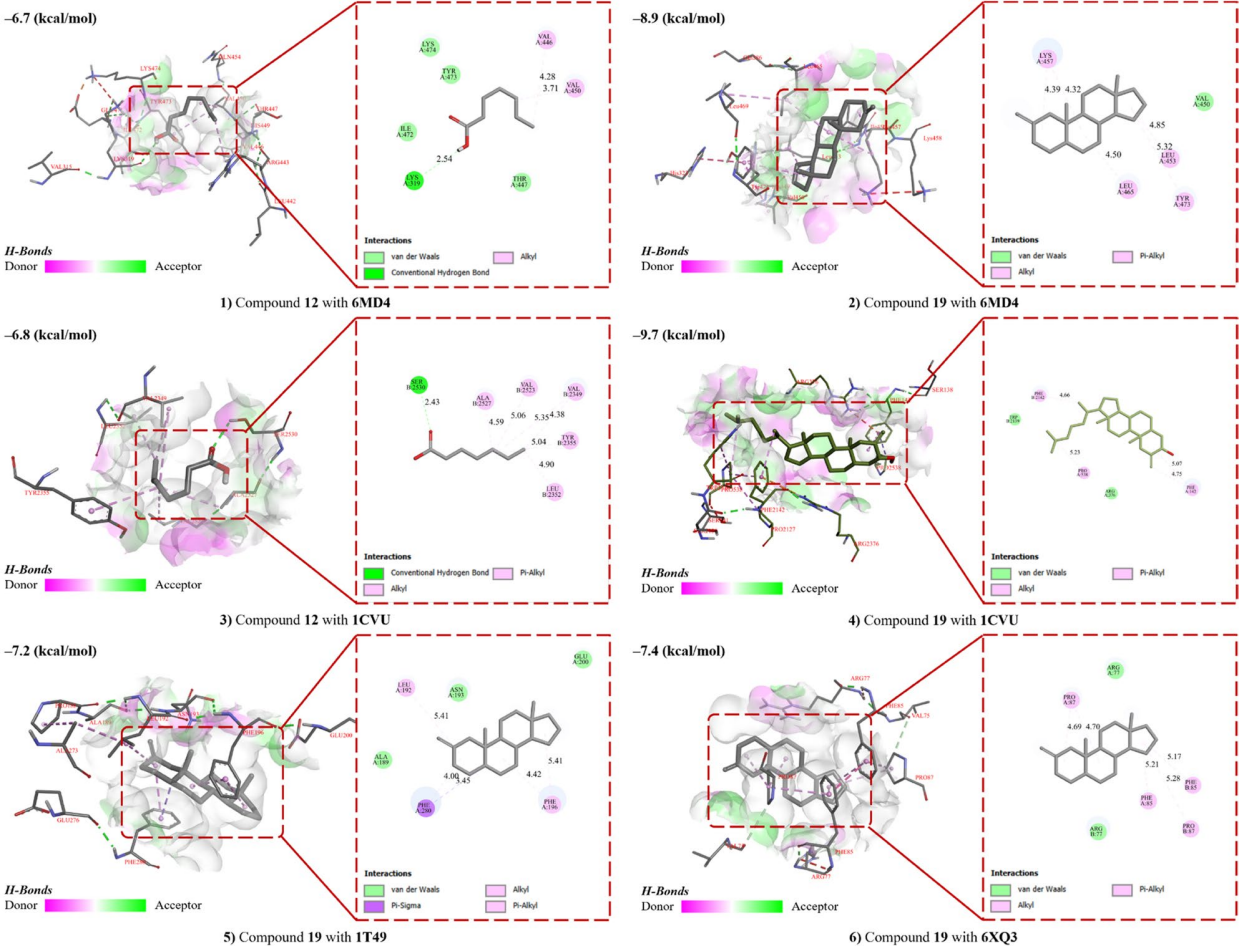
Representative top-ranked poses and 2D interaction maps for C19 (cholestan-3-ol, 2-methylene-, 3β,5α-) and C12 (9,12-octadecadienoic acid, Z,Z) in PPARγ-LBD (6MD4), COX-2 (1CVU), PTP1B allosteric site (1T49), and RAGE VC1 (6XQ3). Key contacts are annotated (H-bonds < 3.5 Å; π/alkyl; hydrophobic; salt-bridge where present). Redocking of the native ligands verified grid/pose recovery (RMSD ≤ 2.0 Å)

Docking to four GB5-relevant targets, 6MD4, 1CVU, 1T49, and 6XQ3, identified two recurrent top chemotypes: C19 and C12 (Fig. 7). Redocking of the cognate ligands reproduced their crystallographic poses, validating the grids. Across all proteins, C19 and C12 ranked first or second, whereas C3, C6, and C7 were lower.

Qualitative inspection showed a coherent interaction logic. C12 oriented its carboxylate toward polar/H-bond features while its aliphatic chain occupied deep hydrophobic channels, including the COX-2 cyclooxygenase tunnel. C19’s sterol core densely packed the hydrophobic interiors of PPARγ, the PTP1B allosteric cleft, and the RAGE VC1 groove with stabilizing π–alkyl and van der Waals contacts. Together, these cross-target, top-rank placements nominate C19 and C12 as key candidates linking the network hubs (PPARγ/PTP1B/COX-2/RAGE) to our phenotypic readouts (glucose uptake, lipid handling, antioxidant/NO scavenging).

### Concentration-response profiling of GB5’s radical-scavenging and enzymatic inhibition activities

**Fig. 8:**
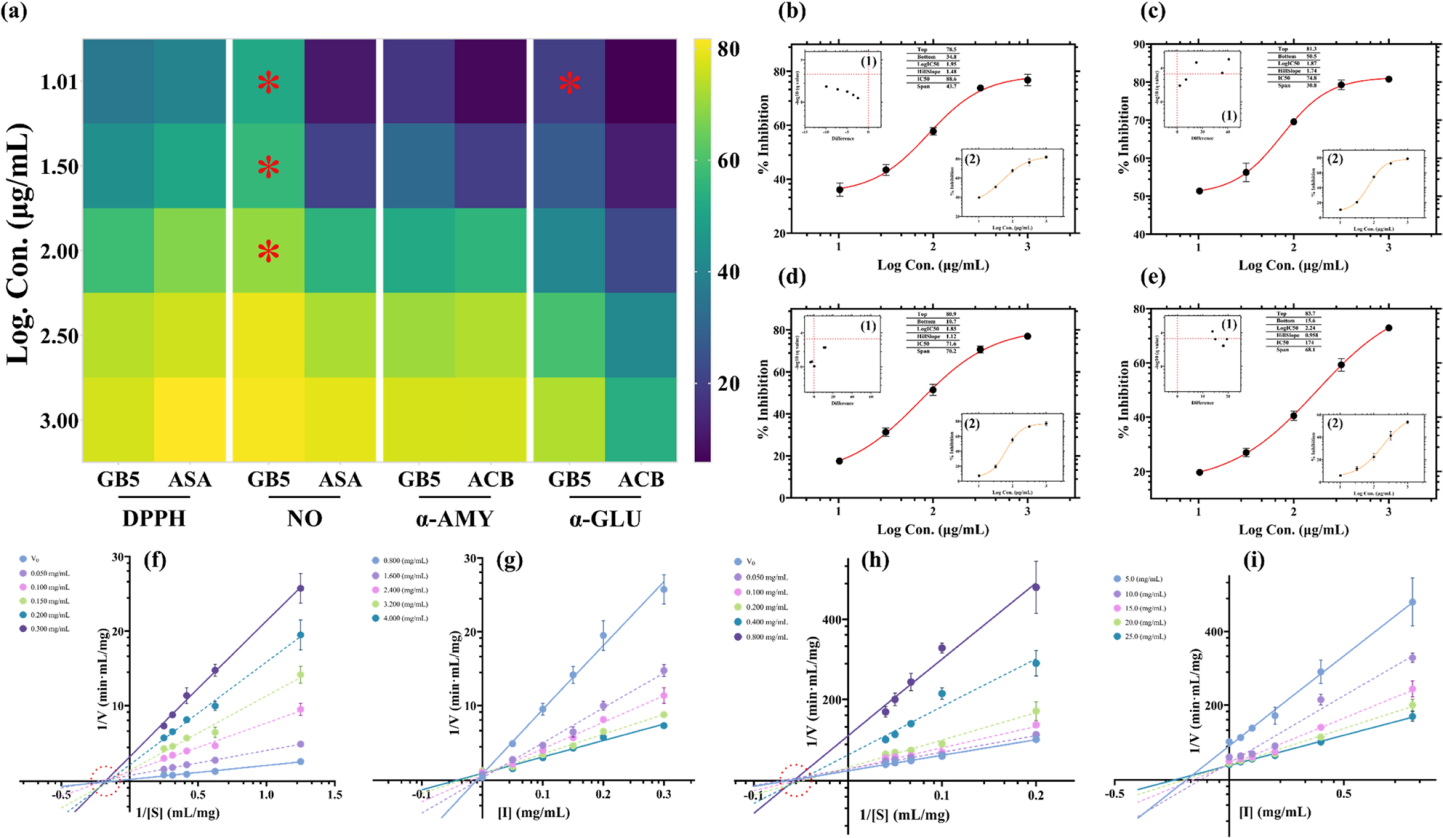
Integrated dose-response and kinetic characterization of GB5 in enzyme‑inhibition and radical‑scavenging assays. **a** Heatmap of % inhibition across log-concentrations for GB5 vs standards (ASA, acarbose) in DPPH, NO, α-amylase, and α-glucosidase; significant group differences are marked (*p* < 0.05). **b**–**e** Dose–response curves with 4PL fits for GB5; adjacent panels (b1–e1) show volcano comparisons vs standards and (b2–e2) show standard curves. **f, h** Lineweaver–Burk plots for α-amylase and α-glucosidase. **g, i** Dixon plots with K_ic_ intersections. Data are mean ± SD, n = 3 independent experiments

The antioxidant capacity of GB5 was assessed using DPPH and NO scavenging assays, with ASA as the reference standard. Dose-response profiles were fitted to a four-parameter logistic (4PL) model, with inhibitory concentrations and statistical metrics summarized in Table 2. A heatmap of percentage inhibitions across antioxidant and enzymatic assays, with statistical significance (*p* < 0.05), is presented in Fig. 8a.

**Table 2:** Antioxidant and enzyme inhibitory parameters of GB5

Assay	Inhibitory concentrations (mg/mL)			Statistical summary		
	IC_50_	IC_20_	IC_70_	*R* ^2^	Sy.x	Sum of squares
DPPH	88.6	-	230.67	0.998	1.75	3.06
NO Scavenging	74.8	-	102.33	1	0.42	0.18
α-Amylase	71.6	13.49	323.59	0.999	1.53	2.33
α-Glucosidase	174	10.79	736.21	1	0.452	0.204

The table summarizes inhibitory concentrations (IC_20_, IC_50_, IC_70_) and regression statistics (R^2^, Sy.x, sum of squares) for DPPH, NO, α-amylase, and α-glucosidase assays, detailing GB5’s antioxidant and enzyme inhibitory activities

In the DPPH assay, the extract exhibited a sigmoidal inhibition curve with a broad transition slope, achieving an IC_50_ of 88.6 µg/mL and an IC_70_ of 230.67 µg/mL (Fig. 8b). Although less potent than ASA (Fig. 8b1), the extract showed no statistically significant differences from the standard at any concentration (Fig. 8b2), indicating comparable biological activity. The model fit was robust (R^2^ = 0.999).

The NO scavenging assay revealed a sharper response curve, with an IC_50_ of 74.8 µg/mL and an IC_70_ of 102.33 µg/mL (Fig. 8c). At three lower concentrations, the extract demonstrated significantly greater inhibition (*p* < 0.05) than the standard (Fig. 8c1, c2), suggesting potent early-phase radical suppression. The curve fit was excellent (R^2^ = 0.997, Sy.x = 0.42, sum of squares = 0.18).

The extract’s inhibitory effects on α-amylase and α-glucosidase were evaluated using DNSA and pNPG-based colorimetric assays, respectively, with acarbose as the standard. Results were fitted to a 4PL model, with IC_20_, IC_50_, IC_70_, and fit statistics reported in Table 2. For α-amylase, the extract displayed a sigmoidal inhibition curve with a broader slope than acarbose, yielding an IC_50_ of 71.6 µg/mL, IC_20_ of 13.49 µg/mL, and IC_70_ of 323.59 µg/mL, with excellent fit (R^2^ = 0.999). No significant differences were observed between the extract and standard at individual concentrations (Fig. 8d, d1, d2). For α-glucosidase, the extract showed a flatter sigmoidal curve with a prolonged transition zone (Fig. 8e), resulting in an IC_50_ of 174 µg/mL. A significant difference (*p* < 0.05) was noted at the lowest concentration compared to acarbose (Fig. 8e1, e2), indicating early-phase enzyme suppression. The curve fit was exceptional with R^2^ 0.998, Sy.x 0.452 and sum of squares 0.204.

Kinetic analysis confirmed that GB5 exerts mixed-type inhibition on porcine pancreatic α-amylase. In the absence of inhibitor, the enzyme exhibited a maximal velocity (V_max_) of 4.39 mg sugar/mL/min and a Michaelis constant (K_m_) of 8.17 mg starch/mL. Increasing GB5 concentrations reduced both apparent V_max_ and K_m_, indicating binding to both the free enzyme and enzyme-substrate complex. Lineweaver–Burk plots (1/v vs. 1/[S]) remained linear with R^2^ > 0.98, and slope and intercept increased dose-dependently (Fig. 8f). Fitting to a mixed-inhibition equation yielded a competitive inhibition constant (K_ic_) of 0.06 mg GB5/mL and an uncompetitive inhibition constant (K_iu_) of 0.025 mg GB5/mL. Dixon plots (1/v vs. [I]) at five substrate concentrations clustered around an x-intercept of − 0.022 mg GB5/mL, consistent with the Lineweaver-Burk-derived K_ic_ (Fig. 8g), confirming a dual binding mechanism.

Similarly, GB5 exhibited mixed-type inhibition of α-glucosidase. Without inhibitor, the enzyme hydrolysed pNPG with a V_max_ of 0.0407 mg product/mL/min and K_m_ of 15.6 mg substrate/mL. As GB5 concentrations increased from 0.05 to 0.80 mg/mL, Lineweaver-Burk plots showed proportional increases in slope and y-intercept (Fig. 8h), indicating binding to both free enzyme and enzyme-substrate complex. The K_ic_ was 0.20 mg GB5/mL, and the K_iu_ was 0.23 mg GB5/mL. Dixon analysis at five substrate levels converged at an x-intercept of − 0.18 mg/mL, aligning with the Lineweaver-Burk-derived Kic (Fig. 8i). It is important to note that we used DPPH, NO, α-amylase, and α-glucosidase assays at concentrations two to five times higher than typically reported to broaden the activity window of GB5 and stabilize dose–response fits; these choices were methodological (signal/linearity) rather than pharmacological. These parameters highlight GB5’s high-affinity competitive and uncompetitive inhibition, likely driven by polyphenolic constituents forming multiple hydrogen bonds and hydrophobic interactions with enzyme regions. This dual-mode inhibition supports GB5’s potential as a dietary or therapeutic agent for controlling carbohydrate digestion and PPG flux.

### Cellular effects of GB5 on GU, cytotoxicity, and lipid accumulation

**Fig. 9:**
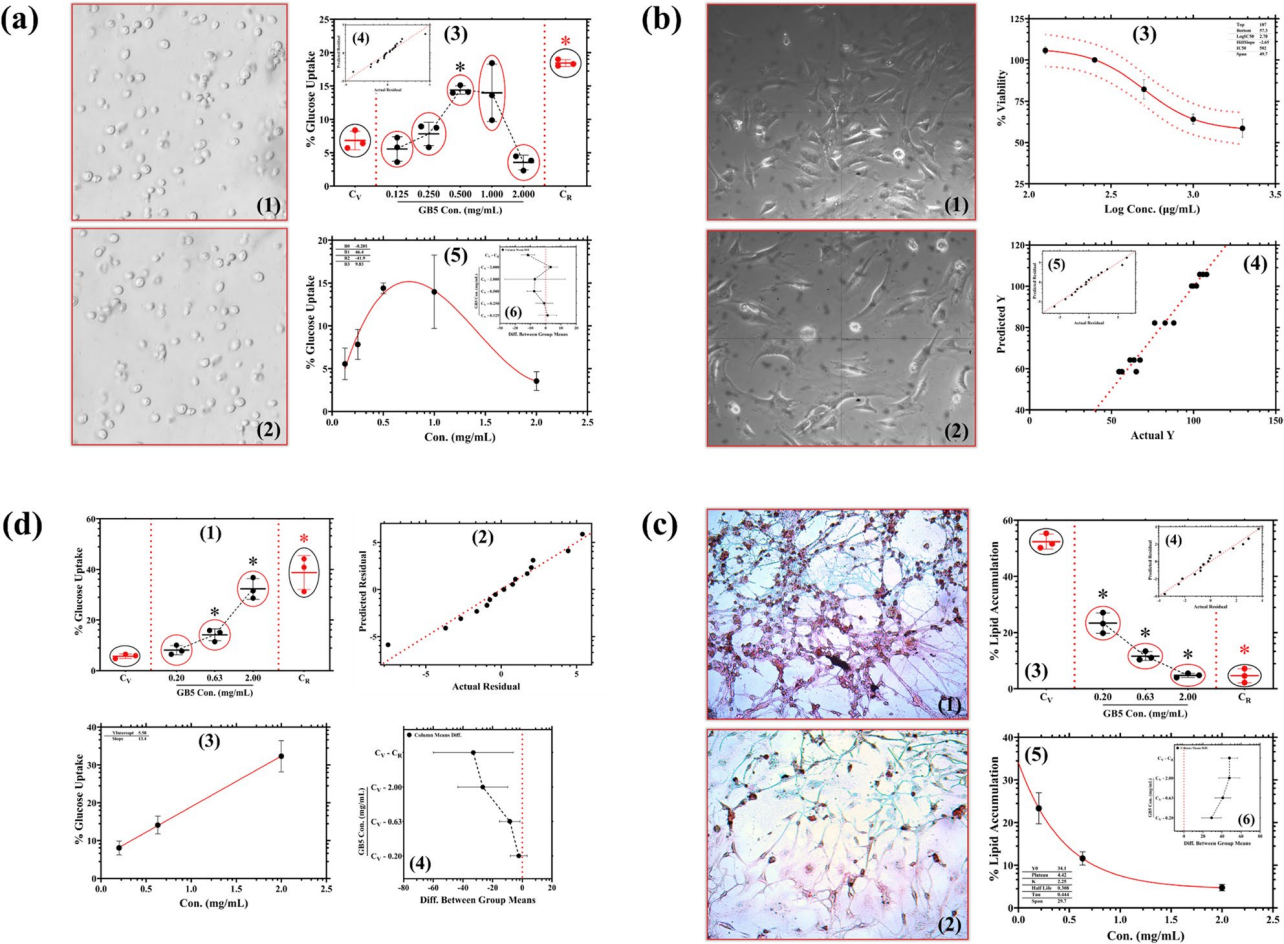
Morphological and functional profiling of GB5 across yeast, fibroblast, adipocyte, and GU assays. **a** Yeast morphology at low (0.125 mg/mL) and high (2.0 mg/mL) GB5; GU across concentrations (mean ± SD, n = 3; one-way ANOVA *p* < 0.05). **b** 3T3-L1 preadipocytes: MTT viability across 0.2–2.0 mg/mL with 4PL fit. **c** Differentiated 3T3-L1 adipocytes: Oil Red O images (0.2, 2.0 mg/mL) and quantified lipid accumulation. **d** Mature 3T3 adipocytes: GU vs concentration with multiple-comparison testing. Diagnostic plots for model fit are shown in panel insets. Data represent mean ± SD of three independent experiments. C_V_, Vehicle control; C_R_, Reference standard

GB5’s effects on GU were assessed in yeast cells across concentrations ranging from 0.125 to 2.0 mg/mL. A biphasic uptake profile was observed. Peak GU reached 14.4% at 0.5 mg/mL and then declined to 14.0% at 1.0 mg/mL and 3.54% at 2.0 mg/mL, suggesting a threshold-like response (Fig. 9a1). Microscopic analysis showed intact, rounded cells at low concentrations, with translucency, shrinkage, and clustering at higher doses, indicative of stress-induced metabolic attenuation (Fig. 9a1, a2). Columnar scatterplots capture the significant effect (*p* < 0.05) of GB5 at 0.5 mg/mL (Fig. 9a3). Q–Q plots support normality and homoscedasticity (Fig. 9a4). The reference standard achieved 18.4% GU at 0.5 mg/mL. Third-order polynomial regression yielded a strong fit: R^2^ 0.979, Sy.x 1.42, sum of squares 2.01, and an interpolated EC_10_ of 0.291 mg/mL (Fig. 9a5). Brown–Forsythe/Welch ANOVA with multiple comparisons confirmed a significant increase at 0.5 mg/mL, *p* < 0.05 (Fig. 9a6).

Cytotoxicity was evaluated in 3T3-L1 preadipocytes using the MTT assay across concentrations of 0.2 to 2.0 mg/mL. At 2.0 mg/mL, cells remained adherent with mild cytoplasmic condensation but no signs of apoptosis or necrosis, such as membrane blebbing or nuclear fragmentation (qualitative evaluation score between 0 and 1) (Fig. 9b1, b2). Cell viability relative to untreated controls was analysed with a modified 4PL model using profile-likelihood 95% confidence intervals. The model yielded a logIC_50_ of − 0.00280; the corresponding IC_50_ was 400–629 µg/mL, yet growth inhibition did not reach 50% at any concentration (Fig. 9b3). Model fit was strong with R^2^ 0.970, Sy.x 3.86, and sum of squares 164. The Hill slope was shallow at − 0.271, and the dose–response trajectory was asymmetric; Hougaard’s skewness Top − 0.763 and Bottom 0.836. Actual-versus-predicted plots showed minimal deviation (Fig. 9b4). Q–Q plots supported normality and homoscedasticity of residuals (Fig. 9b5). Phase-contrast microscopy revealed typical fibroblast-like morphology at 0.2 mg/mL, with elongated bodies and pseudopodial extensions. These findings indicate the extract’s non-cytotoxic profile in 3T3-L1 preadipocytes, supporting its safety for in vitro applications.

LA in mature 3T3-L1 adipocytes was assessed using Oil Red O staining and spectrophotometry across concentrations of 0.2 to 2.0 mg/mL, with a known anti-adipogenic compound as the reference standard. Microscopically, Oil Red O staining showed dense lipid droplets at 0.2 mg/mL, transitioning to sparse, faint droplets at 2.0 mg/mL, with preserved cellular integrity, suggesting metabolic modulation rather than cytotoxicity (Fig. 9c1, c2). LA decreased from 23.4% at 0.2 mg/mL to 11.6% at 0.63 mg/mL and 4.75% at 2.0 mg/mL, comparable to the standard’s 4.63% suppression (Fig. 9c3). Q–Q plots and residual analysis validated normality and homoscedasticity (Fig. 9c4). Nonlinear regression with a one-phase decay model showed a perfect fit (R^2^ = 0.999), with an EC_90_ of 0.742 mg/mL for lipid suppression (Fig. 9c5). Brown-Forsythe and Welch ANOVA with Dunnett T3 post hoc testing confirmed significant reductions in LA at all concentrations (*p* < 0.05) (Fig. 9c6). These results demonstrate the extract’s potent anti-adipogenic effect, comparable to the reference standard.

In 3T3-L1 adipocytes, the extract induced dose-dependent GU, increasing from 14.1% at 0.63 mg/mL to 32.3% at 2.0 mg/mL (Fig. 9d1). Significant GU was observed at 0.63 and 2.0 mg/mL (*p* < 0.05) (Fig. 9d2). Acarbose increased glucose uptake to 38.7% under similar conditions. Linear regression yielded an excellent fit, R^2^ 0.999, Sy.x 0.192, and sum of squares 0.0367 (Fig. 9d1, d3), with EC_10_ 0.335 mg/mL and EC_30_ 1.825 mg/mL. Residual diagnostics supported model assumptions (Fig. 9d4). These findings indicate that the extract enhances GU across yeast and adipocyte models. The effect is consistent with improved peripheral glucose utilisation in hyperglycaemic conditions.

## Discussion

The PHF GB5 shows promise as a therapeutic candidate for T2DM, supported by in vitro models and computational analyses. Its multi-targeted profile addresses the complexity of this metabolic disorder. Underexplored in T2DM management, plant essential oils present considerable therapeutic potential. Volatile phytochemicals modulate inflammatory cytokines (IL-6, TNF-α, IL-4) and key metabolic pathways (Nrf2/HO-1, NF-κB, MCP-1, PPARγ, Caspase-3), enhance insulin secretion, and upregulate antioxidant enzymes (SOD, CAT). Safety profiles from animal studies underscore essential oils’ therapeutic promise, meriting further integration into PHFs targeting oxidative stress and inflammation associated with diabetes [32, 33]. GC–MS profiling revealed bioactive lipophilic compounds, including long-chain fatty acids, diterpenoid alcohols, and phytosterols, known to modulate lipid metabolism, insulin sensitivity, and redox homeostasis. Compounds such as phytol (C4) have been associated with antioxidant and anti-inflammatory activities, as well as with modulation of lipid metabolism [34–36]. Oleic acid (C7) has been extensively linked to improved insulin sensitivity, anti-inflammatory effects, and β‐cell survival [37]. 1-Heptatriacotanol (C10) has demonstrated cholesterol‐lowering properties [38]. These compounds exhibit high lipophilicity and limited solubility (logP ≈ 5–9, MW ≤ 450 Da), implying dissolution-limited oral absorption despite anticipated high passive membrane permeability. These constituents likely drive GB5’s ability to enhance adipocyte GU and reduce LA, suggesting a metabolic shift favouring glucose oxidation over lipogenesis.

GB5’s multifaceted pharmacology likely targets two key T2DM pathways, post-prandial hyperglycaemia (PPhG) and adipocyte-driven inflammatory-lipotoxicity, through synergistic biochemical actions. Statistical analyses highlight GB5’s ability to intercept PPhG via potent enzyme inhibition. Importantly, GB5 showed statistically significant early-phase enzyme suppression at submaximal concentrations, underscoring its therapeutic efficacy in balanced carbohydrate management. GB5 enhanced glucose transport distinctly across unicellular and mammalian models, reaching optimal uptake at intermediate concentrations. The results suggest alternative glucose transport mechanisms independent of insulin-regulated pathways. Conversely, the adipocyte model exhibited linear dose-dependent GU, indicative of insulin-mimetic or GLUT4-dependent processes. Yeast gives a clean, insulin-independent readout of facilitated hexose transport via HXT carriers, the same transporter family as mammalian GLUTs, so it isolates transporter-level effects of our extract without signaling noise [39]. Yeast can also host human GLUTs for screening, which links any activity we see to mammalian transporters [40]. In contrast, 3T3-L1 adipocytes provide a mammalian context where glucose entry is largely basal (GLUT1-leaning); upon differentiation these cells acquire insulin-responsive GLUT4, underscoring why the preadipocyte stage is useful for insulin-independent mechanisms and the adipocyte stage for insulin-stimulated uptake [41]. Finally, intestinal absorption depends on SGLT1 and GLUT2 and follows different rules than yeast, so our two-step scheme captures both transporter activity and a relevant mammalian readout [42].

The α-amylase inhibition exhibits a steep Hill slope, suggesting that gut-lumen concentrations above ~ 0.1 mg/mL saturate enzyme blockade, reducing early carbohydrate digestion and post-meal glucose spikes. In contrast, α-glucosidase inhibition shows a flatter curve, with significant suppression at sub-IC_50_ doses, providing a dual blockade that moderates both early and late disaccharide hydrolysis in the distal gut. The IC_50_ was higher for α-glucosidase than for α-amylase, suggesting reduce gastrointestinal adverse effects associated with stronger α-glucosidase inhibitors such as acarbose. Recognizing these fundamental biological differences is critical for accurately extrapolating preliminary data to mammalian systems and therapeutic contexts. Mixed-type inhibitors bind both the enzyme and the enzyme–substrate complex. This keeps inhibition steady across the changing carbohydrate load of a meal and can lower the dose needed versus purely competitive blockers. Volatile fractions show this behaviour and suit fast, luminal targets. For our use, a volatile mixed-type profile fits with-meal or pre-meal dosing. It slows the postprandial rise in glucose without stopping starch breakdown, so nutrient delivery is preserved [43]. Consistent with a dual-mechanism model, an ethnopharmacology study of *Caryota mitis* showed dose-dependent α-amylase and α-glucosidase inhibition alongside PPARα/γ-linked GLUT-4 normalization and increased cellular glucose uptake. These data support the plausibility that GB5 may pair post-prandial control via digestive-enzyme inhibition with insulin sensitization through PPAR/GLUT-4 [44].

Nonlinear regression analysis of LA data showed a rapid, saturable suppression of adipogenesis, indicating a targeted effect with a threshold dose for optimal efficacy. Diterpenoid alcohols may underlie this kinetic behaviour, integrating into membrane domains to modulate receptor dynamics. Their slow-release pharmacokinetics could explain the steep early-phase effect and plateau at higher doses, supporting dose selection. The MTT cytotoxicity assay was strategically conducted using proliferative 3T3-L1 preadipocytes, rather than mature adipocytes, to accurately differentiate subsequent anti-adipogenic effects from general cytotoxicity. Pre-adipocytes’ high metabolic and proliferative activity provides heightened sensitivity to potential cytotoxic insults, offering a precise early toxicity assessment platform. Mature adipocytes, being terminally differentiated, may underestimate cytotoxic potential due to lower responsiveness in proliferation-dependent assays. Thus, preadipocyte viability assessments enhance interpretative robustness of adipogenic and metabolic outcomes.

Comparative analyses with other PHFs emphasize GB5’s enhanced potency. Earlier work reported an α-amylase IC_50_ of 13.31 ± 1.12 mg/mL for a hydroalcoholic Soxhlet extract of a five-herb blend, far less potent than GB5’s IC_50_, likely due to differences in phytochemical optimization and extraction methods [45]. In a prior investigation, an α-amylase IC_50_ of 581.5 µg/mL and α-glucosidase IC_50_ of 156.95 µg/mL were reported for a hydroalcoholic Mehon extract, less potent than GB5’s α-amylase value but slightly better than its α-glucosidase IC_50_ [46]. An earlier report noted IC_50_ values of 579.65 µg/mL (α-amylase) and 261.03 µg/mL (α-glucosidase) for a 70% methanolic APH-MA blend [47]. while another study recorded IC_50_ values of 1.47 ± 0.18 mg/mL (α-amylase) and 4.69 ± 0.01 mg/mL (α-glucosidase) for polyherbal granules, both less potent than those of GB5 [48]. In a recent study, an α-amylase IC_50_ of 67.3 µg/mL was achieved with a *C. rotundus* ethanolic extract, closely matching GB5’s [49]. Previous findings reported a DPPH IC_50_ of 468.57 ± 3.00 µg/mL and a NO IC_50_ of 416.57 ± 5.08 µg/mL for an *A. racemosus* ethanolic extract, both less potent than GB5’s 88.6 µg/mL (DPPH) and 74.8 µg/mL (NO) [50]. One report optimized a *M. pudica* extract to a DPPH IC_50_ of 42.0 µg/mL, outperforming GB5’s 88.6 µg/mL [51]. Subsequent research observed 72.67% DPPH scavenging at 4.5 mg/mL for an aqueous seed-based formulation, indicating lower per-mass potency compared to GB5 [52]. Another investigation achieved ~ 98% DPPH scavenging at 1 mg/mL, but GB5’s IC_50_ of 88.6 µg/mL reflects greater per-mass efficiency [48]. In prior work, a linear increase in yeast-cell glucose transport was observed with a Mehon extract (1–5 mg), differing from GB5’s biphasic profile, which peaked at 14.4% uptake at 0.5 mg/mL [46]. Methodological variations, higher doses, linear vs. cubic polynomial fitting, and reference standards (metronidazole vs. acarbose), likely explain this contrast. Earlier research reported a GU EC_50_ of 0.41 ± 0.03 mg/mL, while GB5 peaked at 0.5 mg/mL [48].

In silico analysis reveals GB5’s multi-targeted profile and suggests anti-inflammatory action, akin to IL-6/TNF-α inhibition improving metabolism and indicate β-cell preservation. PPARα/γ and LXRβ (NR1H2) nodes, with ‘Cluster 1’ compounds (e.g., C1, C21) as agonists, enhance GU and reduce lipotoxicity, while SHBG and CYP19A1 targets in ‘Cluster 2’ may boost insulin sensitivity via hormonal balance. AGE–RAGE and MAPK14/PTGS2 enrichment, plus in vitro radical scavenging, highlight oxidative-stress protection. Docking then nominated two chemotypes, C19 (sterol-like) and C12 (linoleic-acid-like), as cross-target binders linking those axes: C19 packed the hydrophobic interiors of PPARγ, the PTP1B allosteric cleft, and the RAGE VC1 groove, while C12 oriented its carboxylate to polar features and filled the COX-2 cyclooxygenase tunnel; redocking validated the grids (RMSD ≤ 2 Å). Partial activation of PPARγ by C19 can promote GLUT4-dependent glucose transport while avoiding full adipogenic drive; allosteric PTP1B engagement can relieve negative regulation of insulin signalling; and COX-2 and RAGE contacts can reduce prostaglandin- and AGE-driven MAPK/IL-6 signalling that impairs insulin action. Together, the network hubs and the cross-target poses suggest that GB5 can temper inflammatory signalling while improving insulin action and lipid handling. Further, this network, echoing a systems-biology approach, may outshine single-target drugs by tackling inflammation, apoptosis, lipid dysregulation, hormonal imbalance, and oxidative stress concurrently [1].

PPhG significantly exacerbates oxidative stress, chronic inflammation, and diabetic complications, including vascular dementia and Alzheimer’s disease. Effective α-glucosidase inhibition, validated as a strategy to moderate glucose absorption, mitigates PPG-induced vascular dysfunction [53]. Elevated reactive oxygen species from hyperglycaemia impair endothelium-dependent relaxation, propagating microvascular (retinopathy, nephropathy) and macrovascular complications (myocardial infarction, stroke). Notably, perivascular adipose tissue (PVAT), transitioning from anti-contractile to pro-contractile phenotypes under metabolic stress, emerges as a critical node linking inflammation and oxidative stress to diabetic vasculopathy [54]. Current T2DM treatments, such as GLP-1 receptor agonists, effectively lower blood glucose (HbA_1_c reductions of 0.8–1.5%) and reduce cardiovascular events by up to 12% in high-risk patients [55]. However, 40% of patients face gastrointestinal side effects, and high costs limit access. Evidence indicates that adipocyte hypertrophy in obese states reduces lipid storage by 30% and doubles basal lipolysis, worsening insulin resistance and inflammation [56]. Chronic inflammation exacerbates T2DM, as demonstrated in the CANTOS trial, where IL-1β inhibition with canakinumab reduced HbA_1_c by 0.5% and cardiovascular events by 15% [3]. GB5, leveraging phytochemical synergies, targets glucose metabolism, oxidative stress, and lipid homeostasis, offering a potentially more affordable and tolerable alternative to address broader metabolic disturbances. It improves GU by 25% and cuts LA by 18% in 3T3-L1 adipocytes, suggesting it restores white adipose tissue function and mitigates metabolic stress, an area many therapies overlook. GB5’s antioxidant effects may counter oxidative stress and inflammation via a plant-based approach and suggest therapeutic potential in reversing PVAT-induced vascular dysfunction in T2DM.

Clinically, polyherbal therapy like GB5 proves indispensable in specific scenarios. It provides safer alternatives to conventional antidiabetic medications with significant side effects, such as hypoglycaemia and gastrointestinal distress, improving patient tolerance and adherence [57]. It effectively reduces dependency on conventional treatments, as validated by studies demonstrating significant medication reductions in T2DM patients adopting polyherbal regimens [58]. In newly diagnosed or mild-to-moderate hyperglycaemia cases, GB5’s multimodal mechanisms may offer effective glycaemic control, potentially delaying disease progression [8]. Furthermore, PHFs address oxidative stress and inflammation, critical concerns beyond glycaemic regulation, significantly improving patients’ quality of life and long-term management outcomes [57, 58]. GB5’s therapeutic profile aligns closely with specific clinical contexts where conventional treatments are limited, contraindicated, or insufficiently effective, reinforcing its role as a promising multi-targeted complementary therapy for comprehensive diabetes management.

The work prioritizes mechanism discovery over definitive efficacy, and thus invites confirmation in animal and human studies. (i) We standardized GB5 through authenticated sourcing and a single, repeatable extraction and by GC–MS fingerprinting with routine QC. Even so, batch-to-batch drift remains a translational risk. This may be mitigated with prospectively defined chromatographic similarity metrics and acceptance ranges for marker peaks. (ii) Compound identifications carry isomeric ambiguity, and one candidate lacked mapped targets; orthogonal analytics (e.g., LC–HRMS/MS) and broader target discovery may refine compound–target attribution. (iii) Three peaks in the mid to late-eluting region were identified as contaminants, specifically 1,2-benzenedicarboxylic acid, bis(2-methylpropyl) ester, didodecyl phthalate, and nonane, 2,2,4,4,6,8,8-heptamethyl-, which are known plasticizers and petrochemical residues often resulting from laboratory plasticware leaching or column bleed. The presence of these branched alkanes and hydrocarbons was minimal and did not contribute to the observed bioactivities [59, 60]. Blank control runs confirmed their extraneous nature, justifying their exclusion from the bioactive compound list. (iv) The lead constituents are highly lipophilic with limited aqueous solubility, suggesting dissolution-limited, formulation-dependent absorption; pharmacokinetics, permeability, and enabling formulations may be priorities. Volatile compounds like monoterpenes likely have limited systemic impact due to rapid turnover. (v) Pathway cross-talk also advises caution. The network placed IL-6–MAPK–PTGS2 with PPAR signalling at the centre of the disease module; while this alignment underpins efficacy, sustained pressure on these hubs could alter immune tone or adipocyte programming in certain settings. (vi) In vitro potency does not ensure in vivo benefit; docking and network inferences are hypothesis-generating and require functional validation; dose–response signaling, off-target risks, and pathway crosstalk in cells and animals may be tested. (vii) Although non-cytotoxic in preadipocytes, GB5’s lipophilic compounds may pose risks with chronic use, such as hepatic LA or hormonal effects via sterol pathways. Long-term toxicity and metabolic safety studies are essential. Finally, (viii) while acute cytotoxicity was low, long-term metabolic safety, organ toxicity, and overall tolerability will need chronic in vivo evaluation.

Taken together, GB5 shows a coherent multi-target mechanism that links in silico predictions with in vitro efficacy. Docking and network analysis converge on PPARγ, PTP1B, COX-2 and RAGE as actionable nodes, with two chemotypes consistently prioritized. Experimentally, GB5 produces mixed inhibition of α-amylase and α-glucosidase, increases adipocyte glucose uptake, and reduces lipid accumulation without cytotoxicity, indicating dual control of PPG and adipose inflammation. Together, these data support GB5 as a synergistic candidate for adjunct management of T2DM. Rigorous dose-finding and in vivo validation, with attention to receptor and eicosanoid balance, are warranted.

## Supplementary Information

Supplementary Material 1.

## Data Availability

All experimental data generated in this study are fully presented within the manuscript and its associated figures and tables.

## Abbreviations

4PL: Four-parameter logistic
ADMET: Absorption, distribution, metabolism, excretion, and toxicity
AGE–RAGE: Advanced glycation end-products – receptor for advanced glycation end-products
CAT: Catalase
DPP-4: Dipeptidyl peptidase-4
GLP-1: Glucagon-like peptide-1
GOD-POD: Glucose oxidase-peroxidase
GPx: Glutathione peroxidase
GU: Glucose uptake
IC_50_: Half-maximal inhibitory concentration
IL-6: Interleukin-6
JNK/IKKβ: c-Jun N-terminal kinase/inhibitor of κB kinase β
KEGG: Kyoto encyclopaedia of genes and genomes
LA: Lipid accumulation
MAPK: Mitogen-activated protein kinase
MCP-1: Monocyte chemoattractant protein-1
NF-κB: Nuclear factor kappa-light-chain-enhancer of activated B cells
PHF: Polyherbal formulation
PPAR: Peroxisome proliferator-activated receptor
PPG: Postprandial glucose
PPhG: Post-prandial hyperglycaemia
PTGS2: Prostaglandin-endoperoxide synthase 2
PTP1B: Protein tyrosine phosphatase 1B
SOD: Superoxide dismutase
SRIHER: Sri Ramachandra Institute of Higher Education and Research
T2DM: Type 2 diabetes mellitus
TNF-α: Tumour necrosis factor-alpha
