# Supplementary material for: GB5, a synergistic phytotherapy for type 2 diabetes mellitus management: an integrated polyherbal approach from phytochemical profiling to network pharmacology
Source: BMC Complement Med Ther. 2025 Dec 11;26:16. doi: 10.1186/s12906-025-05192-3 (PMC12801877; doi:10.1186/s12906-025-05192-3)
Supplement: Supplementary file 1 — Supplementary Material 1. [file 12906_2025_5192_MOESM1_ESM.pdf]

Supplementary Material

Supplementary Material for:

GB5, a synergistic phytotherapy for type 2 diabetes mellitus management: An integrated polyherbal approach from phytochemical profiling to network pharmacology

Arghadip Das[1,a], Narmadha Chinnadurai Rajeswari[1,b], Navaneedhakrishnan Manigandan[1,c], Priyadharshini Palani[1,d], Sujatha Kuppusamy[1,e], Gayatri Sukumaran[1,f], Gayathri Veeraraghavan[1,g,*], Raman Lakshmi Sundaram[1,h,*]

[1]Sri Ramachandra Institute of Higher Education and Research, Chennai, Tamil Nadu 600116, India

ORCIDs: 0000–0002–5717–1498[a], 0009–0001–1459–9216[b], 0009–0004–9789–0798[c], 0009–0003–2217–7448[d], 0000–0001–6130–5412[e], 0000–0002–8995–7882[f], 0000–0001–9764–0140[g], 0000–0001–7013–8732[h]

*Address for correspondence:

**Dr. Raman Lakshmi Sundaram, Ph.D.**	**Dr. Gayathri Veeraraghavan, Ph.D.**
Associate Professor – Research<br>Sri Ramachandra Faculty of Pharmacy,<br><br>Sri Ramachandra Institute of Higher Education and Research (DU), Chennai, Tamil Nadu 600116, India<br>E-mail: sundaram@sriramachandra.edu.in<br>Contact No.: (+91) 96000 28292	*HOD / Test facility management (TFM),*<br>Centre for Toxicology and Developmental Research (CEFTE),<br>Sri Ramachandra Institute of Higher Education and Research (DU), Chennai, Tamil Nadu 600116, India<br>E-mail: gayathriv@sriramachandra.edu.in<br>Contact No.: (+91) 44238 60464

Contents

1.0 Table S1 3

2.0 Experimental 4

2.1 Materials S1 4

2.2 Methods S1 4

2.2.1 GC-MS analysis 4

2.2.2 Network pharmacology 5

2.2.2.1 Construction of compound-target network of GB5 5

2.2.2.2 Construction disease-targets protein-protein interaction network 5

2.2.2.3 Network analysis 6

2.2.3 Statistical analysis 6

3.0 *Table S2* 8

References 9

1.0 *Table S1: Pharmacological attributes of selected botanicals individually and within PHFs supporting their inclusion in GB5*

The selection of these botanicals for GB5 is carefully crafted based on their individually validated pharmacological profiles and proven synergistic efficacy in PHFs, which align precisely with the metabolic improvements demonstrated in our study.

Sl. No.	Botanical name	Antidiabetic mechanisms			Associated metabolic actions			Additional relevant pharmacological effects	Clinical validation as part of polyherbal formulations	Clinically proven outcomes	References
		Carbohydrate digestion enzyme inhibition		Glycaemic and insulin-related effects	Antioxidant activity	Anti-inflammatory effects	Lipid metabolism regulation				
		α-GLU	α-AMY								
01	*Asparagus racemosus*	✓	✓	↑ insulin secretion (isolated pancreas), ↓ blood glucose	✓DPPH, ✓OH	↓TNF-α, ✓NO	↓TC, ↓LDL	Mild diuretic; Reduction of polyuria, polydipsia, polyphagia, fatigue	Yes	↓HbA_1c, ↓FG, ↓PPG, ↑Fasting insulin, improved β-cell function (HOMA-B), ↓Insulin resistance (HOMA-IR), safe and tolerable	[1–5]
02	*Cyperus rotundus*	-	✓	↓DPP-4, ↓PTP1B, ↑GLUT1 mRNA, ↑PGU, ↓FG, ↑HG	↑SOD, ↑GPx, ↑CAT, ↓H_2O_2, ✓DPPH	↓TNF-α, ↓IL-6, ↓HAase, ↓MPO	↓Lipase, ↓TG, ↓TC, ↓HDL, ↓LDL, ↓LPO	-	Yes	↓FG, ↓PPG, ↓HbA_1c, safe and tolerable	[6–10]
03	*Tinospora cordifolia*	✓	✓	↓FG, ↑PGU, ↑HG, histopathological protection of β-cells and liver	↑SOD, ↑GSH, ✓DPPH	↓VEGF, ↓PKC, ↓TNF-α, ↓IL-1β, ↓MPO, ↓HAase, ✓NO	↓TG, ↓LDL, ↓LPO	osmotic diuresis	Yes	Reduced diabetes progression, ↓FG, ↓PPG, ↓HbA_1c, ↓PPG, ↓insulin resistance, safe and tolerable	[11–15]
04	*Terminalia arjuna*	✓	-	↓DPP-4, ↓FG, ↓HbA_1C	✓DPPH, ↑CAT, ↑SOD, ↑GSH	↓IL-6, ↓IL-1β, ↓TNF-α, ✓NO	↓TG, ↓TC, ↓HDL, ↓LDL, ↓LPO	Significant reductions in polyphagia, polydipsia, polyuria, nocturia	Yes	↓FG, ↓PPG, ↓HbA_1c, safe and tolerable	[10, 16–18]
05	*Mimosa pudica*	-	✓	2-NBDG uptake, ↓pancreatic insulin, normalized PPG, ↓HbA_1C	↑SOD, ↑GSH, ↑GPx, ↑GST, ✓DPPH	↓IL-1β, ↓TNF-α, ✓NO	↓LPO	Restored vitamin E and C; ↓AST, ↓ALT, ↓ALP, ↓ACP, ↓γ-GTP	Yes	↓FG, ↓PPG, ↓HbA_1c, safe and tolerable	[19–22]

FG, Fasting glucose; **PGU,** Peripheral glucose utilization; **HG,** Hepatic glycogen; **PPG,** Postprandial glucose; **OH,** Hydroxyl radical scavenging potential; **H_2O_2,** Hydrogen peroxide scavenging ability; **TC,** Total cholesterol; **TG,** Triglyceride; **LPO,** Lipid peroxidation.

2.0 Experimental

2.1 Materials S1

IBMX (3-isobutyl-1-methylxanthine) (≥ 98% purity), Porcine Pancreatic α-Amylase (≥ 1000 U/mg protein), Soluble Potato Starch, Acarbose (≥ 95% purity), α-Glucosidase from *S. cerevisiae* (≥ 100 U/mg protein), PAHBAH (p-hydroxybenzoic acid hydrazide) (≥ 97% purity), pNPG (p-nitrophenyl-α-D-glucopyranoside) (≥ 98% purity), MTT (3-(4,5-Dimethyl-2-thiazolyl)-2,5-diphenyl-2H-tetrazolium bromide), Metronidazole (≥ 98% purity), Rosiglitazone (≥ 98% purity), and Dexamethasone (≥ 97% purity) were sourced from Sigma-Aldrich, India. DMEM (Dulbecco's Modified Eagle Medium, high glucose) and Fetal Bovine Serum (FBS, heat inactivated) was procured from Gibco™, USA. DPPH (2,2-diphenyl-1-picrylhydrazyl) (95% purity), L-Ascorbic acid (99.7% purity), Sodium Nitroprusside Dihydrate (99% purity), DNSA (3,5-dinitrosalicylic acid) (99% purity), Insulin (25 USP U/mg), and Oil Red O (75%) was procured from Sisco Research Laboratories Pvt. Ltd., India. All other chemicals were sourced commercially and were of analytical grade.

2.2 Methods S1

2.2.1 GC-MS analysis

GC-MS analysis of the polyherbal extract was conducted using a Clarus 680 gas chromatography system coupled to a Clarus 600 mass spectrometer (PerkinElmer, USA), operating under electron ionization (EI) conditions. System operation and data acquisition were managed through TurboMass™ software, version 5.4.2, with compound identification performed using the NIST mass spectral library (version 2008). For analytical preparation, 2 g of the lyophilized extract was dissolved in 10 mL of ethanol with gentle agitation. The solution was filtered through a 0.45 µm Millex™ hydrophilic PTFE syringe filter (SLFH1R00N, Merck, Germany) to remove particulate matter and higher molecular weight non-volatile constituents. The filtrate was subsequently passed through a column packed with anhydrous Na_2SO_4, and the eluate collected. The eluate was concentrated to a final volume of 1 mL under a gentle nitrogen stream at ambient temperature (25°C). Chromatographic separation was performed using an Elite-5MS capillary column (30 m × 0.25 mm internal diameter × 0.25 µm film thickness) consisting of 5% diphenyl and 95% dimethylpolysiloxane, with helium as

the carrier gas at a constant flow rate of 1.0 mL/min. Injector temperature was maintained at 260°C, and 1 µL of the concentrated extract was injected in split mode (ratio 10:1). The oven temperature program began at 60°C (held for 2 min), ramped at a rate of 10°C/min up to 300°C, and was held isothermally at 300°C for an additional 6 min, yielding a total runtime of 32 min. Mass spectrometry parameters included ion source temperature set at 240°C, interface (transfer line) temperature at 240°C, and a solvent delay of 2 min. Acquisition was carried out in full-scan mode over a mass range of 50–600 m/z, with a scan time of 0.2 sec and scan interval of 0.1 sec. Analytical reproducibility was confirmed through triplicate injections, demonstrating consistent retention times and peak area reproducibility. Routine solvent and matrix blank injections were also conducted to ensure baseline stability and identify potential contaminants such as plasticizers from laboratory consumables or column bleed artifacts, thereby validating peak authenticity.

2.2.2 Network pharmacology

2.2.2.1 Construction of compound-target network of GB5

Network pharmacology analysis was executed to uncover potential therapeutic mechanisms. SDF files of selected compounds were analysed using SwissTargetPrediction (http://www.swisstargetprediction.ch/), leveraging similarity-based algorithms linking chemical structure and bioactivity profiles. Human-specific targets with prediction probabilities ≥ 10% were selected, yielding annotated proteins with UniProt identifiers.

2.2.2.2 Construction disease-targets protein-protein interaction network

Disease-associated genes for ‘Diabetes Mellitus’ and ‘Lipid Metabolism’, retrieved from GeneCards (https://www.genecards.org/) with relevance scores ≥ 10, ensured biological significance. Common targets between compound-associated and disease-related gene sets were identified and analysed using STRING (https://string-db.org/) to construct a high-confidence protein-protein interaction network. For STRING, we selected *Homo sapiens* and kept only experimentally supported or database-curated interactions (evidence mode), requiring a confidence score ≥ 0.70. Functional enrichment analysis, encompassing Gene Ontology biological processes and KEGG, WikiPathways, and Reactome pathways, ranked enriched pathways by false discovery rate. The top 5–10 pathways were visualized using ChiPlot (https://www.chiplot.online/).

2.2.2.3 Network analysis

Interaction networks were further constructed to delineate compound-target-pathway relationships. The CytoHubba plug-in (ver. 0.1) within Cytoscape (ver. 3.10.3) employed the MCC algorithm to identify hub genes based on node degree, centrality, and shortest path metrics. Functional modules within the compound-target network were identified through MCODE analysis (degree cutoff 10, node density ≥ 0.5, node score ≥ 0.5, K-core = 2, max depth = 100). The original STRING network underwent topological analysis via stringApp (ver. 2.2.0) to identify top hub genes essential to network integrity. Visualizations were produced with yFiles Layout Algorithms (ver. 1.1.5) and annotated using the Legend Creator plug-in (ver. 1.1.7) for clarity.

2.2.3 Statistical analysis

All statistical analyses were conducted using GraphPad Prism version 8.0.2 (263) (GraphPad Software, San Diego, CA, USA), supplemented by Microsoft Excel 2021. Data normality was assessed via the Shapiro–Wilk test ($\alpha = 0.05$).

Nonlinear sigmoidal four-parameter logistic (4PL) regression was applied to antioxidant, enzymatic inhibition, and MTT assays to determine EC_{50}/IC_{50} values, with the X-axis log-transformed, following:

$$Span = Top - Bottom \qquad \text{S. Eq. 1}$$

$$Y = Bottom + \frac{Span}{\left(1+10^{\left((\log IC_{50} - X)\cdot HS\right)}\right)} \qquad \text{S. Eq. 2}$$

Here, '*Y*' represents percentage inhibition, '*X*' is log concentration, '*Top*' and '*Bottom*' are asymptotes, '$logIC_{50}$' is log of half-maximal inhibitory concentration, and '*HS*' is the Hill slope. The model fitting employed least squares regression (max 1000 iterations), without weighting. Model suitability for datasets was confirmed by the Extra Sum-of-Squares F test. Confidence intervals (95%) and prediction bands (MTT assay) were plotted, with model fit quality assessed by R^2, Sy.x, and sum of squares.

Enzyme kinetics parameters were derived using linear regression analyses (Lineweaver–Burk and Dixon plots). Velocity data are expressed as mean ± SD from triplicated independent experiments. Initial-rate linearity was confirmed ($R^2 \geq 0.99$) with residual distribution checks. Each regression provided slope, y-intercept (± SE), 95% CI, R^2, Sy·x, and F-statistics.

Lineweaver–Burk mixed-inhibition was evaluated using:

$$\frac{1}{v} = \frac{K_m}{V_{max}}\left(1 + \frac{[I]}{K_{ic}}\right)\frac{1}{S} + \frac{1}{V_{max}}\left(1 + \frac{[I]}{K_{iu}}\right) \quad \text{S. Eq. 3}$$

Dixon plot analyses followed:

$$\frac{1}{v} = \left(\frac{K_m}{[S]\cdot V_{max}\cdot K_{ic}} + \frac{1}{V_{max}\cdot K_{iu}}\right)[I] + \left(\frac{K_m}{V_{max}\cdot [S]} + \frac{1}{V_{max}}\right) \quad \text{S. Eq. 4}$$

Here, 'v' denotes initial velocity, '*[S]*' substrate concentration, '*[I]*' inhibitor concentration, 'K_m' Michaelis constant, 'V_{max}' maximal velocity, 'K_{ic}' competitive inhibition constant, and 'K_{iu}' uncompetitive inhibition constant.

Yeast GU EC_{50} was estimated via cubic polynomial nonlinear regression:

$$Y = B_0 + B_1X + B_2X^2 + B_3X^3 \quad \text{S. Eq. 5}$$

In this equation, 'Y' is percentage GU, 'X' the concentration, and coefficients 'B_0–B_3' are polynomial parameters. Outlier analysis utilized Q = 1%, with robustness evaluated by R^2, Sy.x, sum of squares, and Runs test.

Differentiated 3T3 adipocyte GU EC_{50} employed linear regression:

$$Y = mX + C \quad \text{S. Eq. 6}$$

'Y' represents GU percentage, 'X' concentration, 'm' the slope, and 'C' the intercept, applying identical analytical settings as in the 4PL model.

LA assay in 3T3 adipocytes utilized a one-phase exponential decay nonlinear regression:

$$Y = (Y_0 - Plateau)\cdot e^{-KX} + Plateau \quad \text{S. Eq. 7}$$

Here, 'Y' denotes LA percentage, 'X' formulation concentration, 'Y_0' initial response, '*Plateau*' minimum response, and 'K' decay constant.

Statistical significance for enzymatic inhibition and antioxidant assays was evaluated using multiple unpaired t-tests with Welch's correction, controlling the false discovery rate (FDR) at 0.05 (Benjamini, Krieger, Yekutieli method). GU and LA assays underwent one-way ANOVA (Brown–Forsythe and Welch correction), with post-hoc comparisons by Dunnett's T3 test. Statistical significance was set at $p < 0.05$.

3.0 ***Table S2:*** *Qualitative phytochemical analysis of GB5*

The table presents results from standardized tests on the polyherbal extract, indicating the presence (+, ++, +++) or absence (–) of major phytochemical classes. This profile summarizes the bioactive constituents contributed by the five constituent botanicals.

S. No.	Test	Inference
1	Alkaloids	+++
2	Carbohydrates	+
3	Glycosides	–
4	Proteins and amino acids	+++
5	Flavonoids	++
6	Phenolic compounds	+++
7	Tannins	+++
8	Saponins	–
9	Phytosterol	++
10	Triterpenoid	–
11	Quinones	–
12	Anthraquinone	–
13	Coumarins	+
14	Fixed oils	+

Relative abundance of the phytochemical groups denoted as '+', low; '++', moderate; '+++', high; and '–', absent.

References

1. Karuna DS, Dey P, Das S, Kundu A, Bhakta T. In vitro antioxidant activities of root extract of Asparagus racemosus Linn. J Tradit Complement Med. 2018;8:60–5. https://doi.org/10.1016/j.jtcme.2017.02.004.
2. Vadivelan R, Gopala Krishnan R, Kannan R. Antidiabetic potential of Asparagus racemosus Willd leaf extracts through inhibition of α-amylase and α-glucosidase. J Tradit Complement Med. 2019;9:1–4. https://doi.org/10.1016/j.jtcme.2017.10.004.
3. Akhtar S, Gupta AK, Naik B, Kumar V, Ranjan R, Jha AK, et al. Exploring pharmacological properties and food applications of Asparagus racemosus (Shatavari). Food Chem Adv. 2024;4 April 2023:100689. https://doi.org/10.1016/j.focha.2024.100689.
4. Pandya S, Savaliya C, Thummar K, Gothwad A, Panchabhai T, Nagore D. Validation of standardized polyherbal formulation in the management of type 2 diabetes mellitus: A randomized, double-blind, placebo-controlled trial. J Diabetes Metab Disord. 2023;22:495–506. https://doi.org/10.1007/s40200-022-01171-4.
5. Plangsombat N, Rungsardthong K, Kongkaneramit L, Waranuch N, Sarisuta N. Anti-inflammatory activity of liposomes of Asparagus racemosus root extracts prepared by various methods. Exp Ther Med. 2016;12:2790–6. https://doi.org/10.3892/etm.2016.3661.
6. Pirzada AM, Ali HH, Naeem M, Latif M, Bukhari AH, Tanveer A. Cyperus rotundus L.: Traditional uses, phytochemistry, and pharmacological activities. J Ethnopharmacol. 2015;174:540–60. https://doi.org/10.1016/j.jep.2015.08.012.
7. Gopalakrishna Pillai GK, Bharate SS, Awasthi A, Verma R, Mishra G, Singh AT, et al. Antidiabetic potential of polyherbal formulation DB14201: Preclinical development, safety and efficacy studies. J Ethnopharmacol. 2017;197:218–30. https://doi.org/10.1016/j.jep.2016.07.062.
8. Abdella FIA, Toumi A, Boudriga S, Alanazi TYA, Alshamari AK, Alrashdi AA, et al. Antiobesity and antidiabetes effects of Cyperus rotundus rhizomes presenting protein tyrosine phosphatase, dipeptidyl peptidase 4, metabolic enzymes, stress oxidant and inflammation inhibitory potential. Heliyon. 2024;10:e27598. https://doi.org/10.1016/j.heliyon.2024.e27598.

9. Awasthi H, Nath R, Usman K, Mani D, Khattri S, Nischal A, et al. Effects of a standardized Ayurvedic formulation on diabetes control in newly diagnosed Type-2 diabetics; a randomized active controlled clinical study. Complement Ther Med. 2015;23:555–61. https://doi.org/10.1016/j.ctim.2015.06.005.

10. Kitphati W, Sato VH, Peungvicha P, Saengklub N, Chewchinda S, Kongkiatpaiboon S, et al. Antihyperglycemic activity of a novel polyherbal formula (HF344), a mixture of fifteen herb extracts, for the management of type 2 diabetes: Evidence from in vitro, ex vivo, and in vivo studies. Heliyon. 2024;10:e38703. https://doi.org/10.1016/j.heliyon.2024.e38703.

11. Majhi S, Singh L, Verma M, Chauhan I, kumari R, Sharma M. In-vivo evaluation and formulation development of polyherbal extract in streptozotocin-induced diabetic rat. Phytomedicine Plus. 2022;2:100337. https://doi.org/10.1016/j.phyplu.2022.100337.

12. Sharma R, Amin H, Galib, Prajapati PK. Antidiabetic claims of Tinospora cordifolia (Willd.) Miers: Critical appraisal and role in therapy. Asian Pac J Trop Biomed. 2015;5:68–78. https://doi.org/10.1016/S2221-1691(15)30173-8.

13. Telapolu S, Kalachavedu M, Punnoose AM, Bilikere D. MD-1, a poly herbal formulation indicated in diabetes mellitus ameliorates glucose uptake and inhibits adipogenesis - An in vitro study. BMC Complement Altern Med. 2018;18:1–11. https://doi.org/10.1186/s12906-018-2177-x.

14. Choudhari VP, Gore KP, Pawar AT. Antidiabetic, antihyperlipidemic activities and herb–drug interaction of a polyherbal formulation in streptozotocin induced diabetic rats. J Ayurveda Integr Med. 2017;8:218–25. https://doi.org/10.1016/j.jaim.2016.11.002.

15. Nakanekar A, Kohli K, Tatke P. Ayurvedic polyherbal combination (PDBT) for prediabetes: A randomized double blind placebo controlled study. J Ayurveda Integr Med. 2019;10:284–9. https://doi.org/10.1016/j.jaim.2018.05.004.

16. Mohanty IR, Borde M, Kumar C S, Maheshwari U. Dipeptidyl peptidase IV Inhibitory activity of Terminalia arjuna attributes to its cardioprotective effects in experimental diabetes: In silico, in vitro and in vivo analyses. Phytomedicine. 2019;57:158–65. https://doi.org/10.1016/j.phymed.2018.09.195.

17. Deshpande S, Jadhav K. Efficacy and safety of Vidangadi Yoga (ayurvedic polyherbal medicine) in type 2 diabetes mellitus: A randomized controlled clinical study. Clin Trials Degener Dis. 2018;3:123.

https://doi.org/10.4103/2542-3975.248011.

18. Cota D, Mishra S, Shengule S. Beneficial role of Terminalia arjuna hydro-alcoholic extract in colitis and its possible mechanism. J Ethnopharmacol. 2019;230:117–25. https://doi.org/10.1016/j.jep.2018.10.020.

19. Sasidharan S, Nair A K, R L, Nair AV, SA S, Joseph SG, et al. A randomized multi-arm open labelled comparative clinical trial report of Pankajakasthuri DiabetEaze powder, a novel polyherbal formulation on the nutritional management and glycemic control in type 2 diabetic and prediabetic patients. Heliyon. 2025;11:e42631. https://doi.org/10.1016/j.heliyon.2025.e42631.

20. Thirukkalukundram Singarapriyavardhanan S, Timiri Shanmugam PS, Koppala Narayana SK, Ammari AA, Amran RA, Alhimaidi AR. Mimosa pudica alleviates streptozotocin-induced diabetes, glycemic stress and glutathione depletion in Wistar Albino Rats. J King Saud Univ - Sci. 2022;34:102037. https://doi.org/10.1016/j.jksus.2022.102037.

21. Baharuddin NS, Roslan MAM, Bawzer MAM, Mohamad Azzeme A, Rahman ZA, Khayat ME, et al. Response surface optimization of extraction conditions and in vitro antioxidant and antidiabetic evaluation of an under-valued medicinal weed, Mimosa pudica. Plants. 2021;10. https://doi.org/10.3390/plants10081692.

22. Adurosakin OE, Iweala EJ, Otike JO, Dike ED, Uche ME, Owanta JI, et al. Ethnomedicinal uses, phytochemistry, pharmacological activities and toxicological effects of Mimosa pudica- A review. Pharmacol Res - Mod Chinese Med. 2023;7 March. https://doi.org/10.1016/j.prmcm.2023.100241.
